# Post‐Translational Modified Neoantigens in Autoimmune Diseases: Challenges of Immune Tolerance

**DOI:** 10.1002/advs.202501766

**Published:** 2025-06-19

**Authors:** Yue Zhai, Kefei Wu, Qi Lin, Zhenzhen Cao, Yankun Jia, Ping Zhu

**Affiliations:** ^1^ Department of Clinical Immunology Xijing Hospital and Department of Cell Biology of National Translational Science Center for Molecular Medicine Fourth Military Medical University Xi'an Shaanxi 710032 China; ^2^ State Key Laboratory of New Targets Discovery and Drug Development for Major Diseases Xi'an Shaanxi 710032 China; ^3^ Xijing Innovation Research Institute Fourth Military Medical University Xi'an Shaanxi 710032 China

**Keywords:** autoimmune diseases, autoreactive T cells, neoantigens, post‐translational modifications

## Abstract

Autoimmune diseases, like spondyloarthritis (SpA) and rheumatoid arthritis (RA), have a high incidence and disability rate and are major diseases that seriously endanger human health. The pathogenesis of autoimmune diseases involves the interaction among genetic factors, environmental factors, and immune disorders. The post‐translational modified neoantigens are the key nodal of these three factors. Under the intervention of the external environment, changes in metabolic status in vivo induce metabolite‐related post‐translational modifications (PTMs). These PTM proteins and peptides, after being presented by human leukocyte antigen (HLA) molecules of specific genotypes, can trigger autoreactive T cell expansions which are antigen‐specific and then develop into autoimmunity. Such biological processes are a great challenge to the immune tolerance status and can be an important cause of autoimmune diseases.

## Graphical abstract

Autoimmune diseases have a high incidence and disability rate. The pathogenesis of autoimmune diseases involves the interaction among genetic factors, environmental factors, and immune disorders. The post‐translational modified neoantigens are the key nodal of these three factors. And these post‐translational modified neoantigens, after being presented by HLA molecules, can activate antigen‐specific autoreactive T cells and autoimmunity.

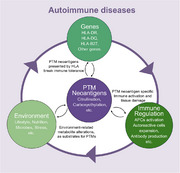

## 1 Introduction

Autoimmune disorders are largely driven by abnormalities in immune tolerance function for self‐recognition.^[^ ^1^ ^]^ Normally the immune system possesses a robust defense function to fight off foreign non‐self antigens. However, when immune tolerance fails, the immune system begins to target the autologous cells. The main causes of damage to these diseases are the series of immune responses probably caused by “false” antigen recognition of normal tissues and organs.^[^ ^2^ ^]^ One of the possible “false” antigen could be induced by pathogen infections, namely molecule mimicry and epitope spread. When susceptible individuals undergo viral or bacterial infection, self‐peptides that resemble viral or bacterial peptides are presented to the major histocompatibility complex (MHC) molecule and cross‐reactive T and B cells are activated. In this case, abnormal autoreactive cell activations or antibody productions triggered by pathogens are key factors. In the meantime, these autoreactive T cells may be specific for epitopes that are not identical to the primary pathogenic epitope, which was termed “epitope spreading” (ES). It is thought that the cross‐reactivity based on molecular mimicry is the initial step to ES and can trigger the initiation of autoimmune response in individuals who are genetically vulnerable.^[^ ^3^ ^]^ Recently, it has been found that the epitope spreading process could cross the MHC barrier in a mixed chimera mouse model,^[^ ^4^ ^]^ and the inclusion of wildtype B cells in chronic autoreactive germinal center propels epitope spreading.^[^ ^5^ ^]^

The other “false” antigens may be originated from the “changed self”, which is more like a non‐self antigen produced by individuals instead of the pathogen infections. These variational peptides may be due to the mutations in the coding amino acid sequence, or the changes in the non‐coding amino acids (ncAAs) which are usually called PTMs. Such post‐translational neoantigens can be generated through the chemical reaction between protein and metabolic products. These modified autoantigens can function as the pivot around normal tissue and initiate autoimmune responses. Altogether, these factors bring up the breakdown of self‐tolerance and trigger the following autoimmunity.

## 2 The Pathogenesis of Autoimmune Diseases: Neoantigen‐Related Immune Disorders

### 2.1 Classical Pathogenic Mechanisms: Environmental Factors, Genetic Factors, and Abnormal Regulation of Immunity

The prevalence of autoimmune diseases is high, and more than 80 different diseases have been identified, such as systemic lupus erythematosus (SLE), ankylosing spondylitis (AS) and rheumatoid arthritis (RA). These diseases are contributing to a rise in the overall direct medical expenses related to autoimmune conditions globally.^[^ ^6^ ^]^ The onset of autoimmune diseases is propelled by the complex interplay among environmental influences, genetic predispositions, and immune system imbalances. Genetic influences are crucial, with specific HLA alleles being closely related to increasing disease risk. The HLA‐DRB1 gene is associated with an increased risk of developing RA,^[^ ^7^ ^]^ while HLA‐B27 is strongly conducive to having a higher risk of AS.^[^ ^8^ ^]^ Environmental factors can further exacerbate the immune response in autoimmune diseases. For example, cigarette smoking and air pollution are identified as major contributors to RA.^[^ ^9^ ^]^ Heavy metal exposure, diet, and other potential chemical stimuli can be environmental pathogenic factors for AS.^[^ ^10^ ^]^ Also, microorganisms in the environment or in symbiosis with the human body can also be used as a source of antigenic mimics in vivo to induce the triggering of self‐reactive T cells and B cells.^[^ ^11^ ^]^

**Figure 1:**
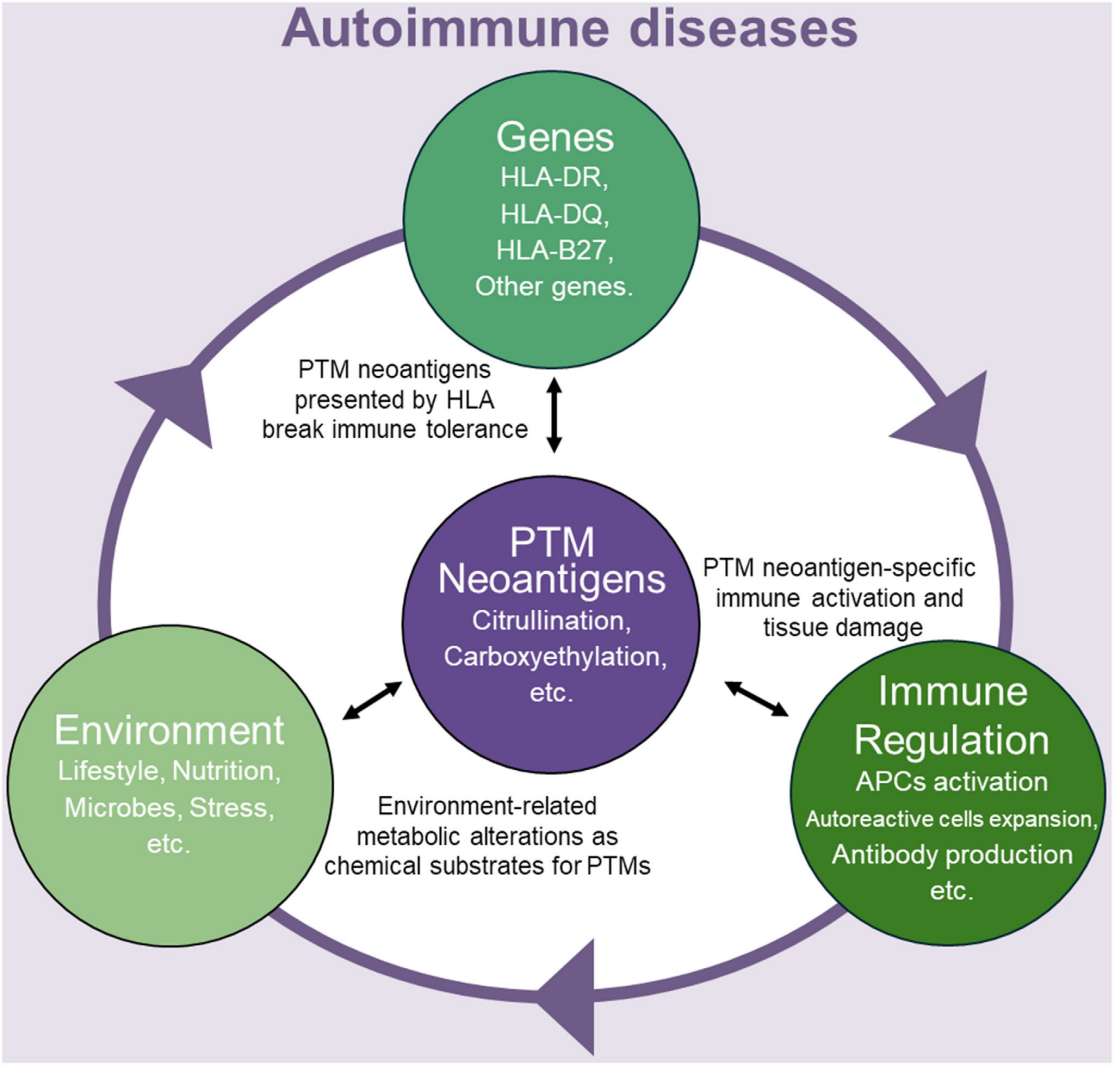
The PTM‐derived neoantigens act as the intersection of environmental factors, genetic factors, and immune dysregulation in autoimmune diseases. The traditional view is that environmental, genetic, and immunomodulatory factors are the three important aspects that induce autoimmune diseases. Post‐translationally modified neoantigens, such as citrullinated or carboxyethylated antigens, can be derived from interactions with the environment, i.e., metabolite changes induced by environmental changes become key metabolic substrates for PTMs. After the above modifications are generated, they can be further presented by the special genotyping of HLA molecules, which provides the possibility of breaking through immune tolerance. On this basis, post‐translationally modified neoantigens can promote the expansion of autoreactive T cells, and produce autoantibodies. Autoantibodies and self‐reactive cytotoxic T cells can immunize and damage the normal tissues and organs where the post‐translational modified antigens are located.

The coordinated influence of environmental and genetic factors can cause autoimmune diseases. Individuals carrying the HLA‐DR4 allele are more susceptible to RA when exposed to smoke,^[^ ^12^ ^]^ which induces citrullination of proteins and produces neoantigens recognized by autoreactive T cells. Similarly, the HLA‐B27 allele associated with AS interacts with the gut microbiota to promote inflammation of the eyes, gut, spine, and sacroiliac joints.^[^ ^13^ ^]^ These gene‐environment interactions highlight the importance of a personalized approach to the prevention and treatment of autoimmune diseases (**Figure** **1**).

In autoimmune conditions, immune system dysregulation is predominantly evidenced by the hyperactivation of autoreactive CD4^+^ and CD8^+^ T cells. Researches reveal that most autoreactive T cells are restricted to class II MHC antigens which are pivotal in autoimmune diseases. An earlier proinflammatory environment with cytokine release by TCR‐independent bystander activation facilitates the upregulation of MHC‐II antigens to optimize antigen presentation, thereby enhancing CD4^+^ T cell activation, while also allowing low‐affinity, rare self‐antigen pMHC complexes to interact with autoreactive TCRs.^[^ ^14^ ^]^ CD4^+^ T cells, particularly the Th1 and Th17 subpopulations,^[^ ^15^ ^]^ play an important part in driving the pathogenesis of autoimmune diseases and inflammatory responses by secreting cytokines like IL‐17 and IFN‐γ. Adoptive administration of Th1 cells is sufficient to elicit the development of EAE in mouse models. IFN‐γ‐induced activation of macrophages and several inflammatory cells contribute to elevated production of pro‐inflammatory cytokines and disruption of homeostatic regulation.^[^ ^16^ ^]^ In the meantime, it's reported that inflammatory response amplifier IL‐17 can also contribute to joint damage in RA through mechanisms involving the induction of tissue‐degrading enzymes, synovial pannus expansion, osteoclast formation, and angiogenesis and induces neutrophil extracellular trap formation, promoting the pathogenesis of SLE.^[^ ^17^ ^]^

CD8^+^ T cells also cause tissue damage in autoimmune diseases by targeting autoantigens presented by MHC class I antigens. It's reported that CD8^+^ tissue‐resident memory T cells are significant contributors to inflammation, with a notable accumulation of type 17 CD8^+^ T cells observed in the joints impacted by psoriatic arthritis.^[^ ^18^ ^]^ In type 1 diabetic patients, islet‐specific self‐reactive CD8^+^ T cells within the pancreas are a characteristic phenomenon that distinguishes them from healthy donors.^[^ ^19^ ^]^

Chronic inflammatory states are also a common disease featured in patients with autoimmune diseases. Such chronic inflammatory states, driven by a complex interplay of cytokines, chemokines, and immune cells, typically arise from an imbalance between anti‐inflammatory and pro‐inflammatory responses. This imbalance is also closely related to the environment and the mutation or activation of inflammatory genes. Cytokines, including interleukin‐6 (IL‐6), and interleukin‐1 beta (IL‐1β), tumor necrosis factor‐alpha (TNF‐α), as well as chemokines like C‐X‐C motif chemokine 16 (CXCL16) and CXCL10, perform vital functions in inflammation by mediating the attraction and stimulation of immune cells. These signaling molecules are crucial for orchestrating inflammatory responses.^[^ ^20^ ^]^ This inflammation not only induces tissue damage, but also leads to system‐wide manifestations of autoimmune conditions, such as exhaustion, fever, and weight loss.^[^ ^21^ ^]^

All three pathogenic aspects have been widely recognized, but in fact, the core intrinsic relationship between the three factors is still unclear. However, recent studies have reported that PTMs may be triggered by metabolites. The modified protein peptides can be recognized as a neoantigen,^[^ ^22^ ^]^ which can cause abnormal immune activation and induce autoimmune diseases after being presented by HLA. Such biological processes can better integrate the three factors of environment, genes, and immune regulation abnormalities into a unified and broader new pathogenic theory.

### 2.2 Neoantigens and Immune Responses

#### 2.2.1 Neoantigens‐Related Adaptive Immune Responses

The concept of neoantigens was first proposed in tumors.^[^ ^23^ ^]^ Most refer to non‐self antigens caused by somatic mutation that are recognized by the immune system, leading to the activation of autoreactive T cells, and providing a new target for tumor immunotherapy.^[^ ^24^ ^]^ Nowadays, neoantigens have expanded from tumors to infectious diseases and autoimmune diseases. At the same time, the source of neoantigens is not only somatic mutations but also PTMs.

The chemical nature of neoantigens is that the primary structure of the antigenic peptide is altered, making it different from the polypeptide library of normal tissue proteins. Alterations in the primary structure of the sequence could induce the immune system to target these peptides as “foreign” that have become neoantigens and cause immune responses. It can be said that neoantigens are a factor that causes the body's immune activation similar to foreign antigens (such as antigens derived from pathogens).^[^ ^25^ ^]^ In cancer, neoantigens result in unique epitopes on the cell surface that are presented by MHC molecules. Such newly appearing antigens are mainly from the genetic instability of tumor cells, such as the broad TP53 mutant increase in epithelial cancers.^[^ ^26^ ^]^ Conversely, by altering the primary structure and sequence of amino acids, microorganisms that infect the host escape the host's immune defense response.^[^ ^27^ ^]^

In the tissues and organs of autoimmune diseases, there are not a large number of somatic mutations like what happen in tumors. Therefore, in the past, it was difficult to screen for neoantigens for autoimmune diseases based on genetic mutations and other related methods. In contrast, PTMs change the primary structure of the amino acid sequence, induce the production of related neoantigens, and provide an important source of neoantigens in autoimmune diseases.

In fact, specific HLA alleles are associated with different autoimmune diseases, strongly suggesting that there may be neoantigens for disease‐associated HLA involved in pathogenesis. These PTM‐change peptides may acquire a stronger ability to bind to the HLA molecule, and the increased affinity is the basis for the presentation of potential antigens by antigen‐presenting cells.

#### 2.2.2 Adaptive Immune Response Disorders Induce Autoimmunity

Although the search for key neoantigens for the disease is difficult, researchers have demonstrated that adaptive immune response disorders, especially the abnormal stimulation of CD4^+^ and CD8^+^ T cells, are pivotal in the onset and progression of autoimmune diseases.

Considered a classic CD4^+^ T cell‐driven disease, RA is featured with inflammation and the production of autoantibodies. Within the synovial tissue of individuals with RA, CD4^+^ T cells are the dominant subset.^[^ ^28^ ^]^ Similar to their synovial counterparts, CD4^+^ T cells in peripheral circulation are capable of recognizing autoantigens and triggering cellular immune responses. The differentiation of naïve T cells into Th1 cells is associated with pro‐inflammatory cytokines production such as TNF‐α, IFN‐γ, and lymphotoxin. This process contributes to the progression of chronic inflammatory diseases and the subsequent degradation of bone and cartilage.^[^ ^29^ ^]^ However, it is also observed that the rheumatoid synovium predominantly contains Th17 cells that produce IL‐17 rather than Th1 cells that produce IFN‐γ.^[^ ^30^ ^]^ In a severe combined immunodeficiency mouse model, the development of arthritis can be observed when primed autoreactive CD4^+^ Th1 cells are transferred and subsequently stimulated with the relevant autoreactive antigen.^[^ ^31^ ^]^ Overall, these findings emphasize the critical role of T cells, especially CD4^+^ T cells, to the autoimmune processes related to RA.

With demyelination and axonal damage, the pathogenesis of multiple sclerosis (MS) is signified by T and B lymphocyte infiltration.^[^ ^32^ ^]^ Autologous myelin‐specific T cells, which are preliminarily triggered in the peripheral, are hypothesized to cross the blood‐brain barrier, and then trigger microglia and macrophages in the brain, secreting IFN‐γ and other pro‐inflammatory cytokines, promoting local inflammation.^[^ ^33^ ^]^ Research has demonstrated that myelin basic protein (MBP)‐specific CD8^+^ T cells can intensify brain inflammation through FasL‐dependent mechanisms.^[^ ^34^ ^]^ To be surprised, the mere introduction of myelin oligodendrocyte glycoprotein (MOG)‐reactive CD8^+^ T cells was sufficient to trigger experimental autoimmune encephalomyelitis (EAE) in mice.^[^ ^35^ ^]^

SLE is an autoimmune disorder with aberrant activity of the immune system, featured by immune tolerance loss and sustained production of autoantibodies. CD4^+^T cells like Th1, Th17, and Th2 cells produce various mediators amplifying the non‐specific immune response, thereby facilitating antibody production and targeted cell lysis.^[^ ^36^ ^]^ In SLE patients with nephritis of III or IV degree, CD8^+^ T cells predominantly infiltrate the kidney and the effector memory phenotype exists in the urine sediment, contributing to tissue damage and their accumulation correlates with the degree of disease activity.^[^ ^37^ ^]^ These infiltrating T cells undergo clonal expansion inferred by the TCR sequence and the same clonotype persists even years later.^[^ ^38^ ^]^ In addition, double‐negative T cells, probably derived from self‐reactive CD8^+^ T cells in tissues, are involved in the SLE pathogenesis because they can also produce IL‐17 and interact with aberrant B cells to augment the production of autoantibody.^[^ ^39^ ^]^

Type 1 diabetes (T1D) is a T cell‐driven autoimmune condition noted for the selective impairment of insulin‐producing β cells by T lymphocytes, succeeded by an autoimmune antibody response.^[^ ^40^ ^]^ This process could lead to insulin deficiency and impaired glucose homeostasis. The islet‐specific self‐reactive CD4^+^ and CD8^+^ T cells have been observed in peripheral blood circulation, pancreatic lymph nodes, and inflamed pancreatic islets.^[^ ^41^ ^]^ A diverse array of autoreactive T cells along with NK cells and macrophages, are triggered and oriented toward mistakenly targeting pancreatic β cells as exogenous agents, resulting in their dysfunction and destruction, impaired insulin secretion and disrupted glucose homeostasis eventually contributing to the development and progression of T1D.^[^ ^42^ ^]^

Inflammatory bowel diseases (IBDs) encompass a spectrum of chronic inflammatory conditions impacting the gastrointestinal tract. Dysregulated immune responses, especially those involving CD4^+^ T cell responses, are considered a driving factor for IBD. A study demonstrated that CD4^+^ T cells reactive to commensal and yeasts originating from food were significantly elevated in peripheral blood and sites of inflammation in CD patients.^[^ ^43^ ^]^ Conversely, the function of CD8^+^ T cells remains a subject of debate. Several researches suggest that CD8^+^ T cells possess anti‐inflammatory properties in the context of colitis,^[^ ^44^ ^]^ while others indicate their involvement in promoting tissue inflammation.^[^ ^45^, ^46^ ^]^

Celiac disease (CeD), classified as a gluten‐mediated intestinal disorder, represents a distinct intestinal condition with autoimmune‐like features that manifest following the ingestion of cereal gluten proteins. In celiac lesion, antibodies targeting gluten and the autoantigen tissue transglutaminase 2 (TTG) exhibit high disease specificity and accumulate prominently,^[^ ^47^ ^]^ alongside gluten‐reactive CD4^+^ T cells restricted by HLA‐DQ2 or HLA‐DQ8. These gluten‐reactive CD4^+^ T cells are predominantly effector memory phenotype,^[^ ^48^ ^]^ which are overlapping clonotypes in individuals after more than 20 years apart. TTG can post‐translationally modify gluten peptides through deamidation, converting specific glutamine residues to glutamate residues with a negative charge. This modification enhances how well the peptides bind to HLA‐DQ2 or HLA‐DQ8 molecules.^[^ ^49^ ^]^

Taken together, it is not difficult to see that neoantigens may be a key link in autoimmune diseases. Comprehensive research on the origins, generation, and immunological effects of neoantigens in autoimmune diseases will play a vital role in improving our understanding and revealing the pathogenic processes involved in these immune‐related conditions. Among them, PTMs will be one of the most important potential sources of neoantigen in autoimmune diseases (Figure 1).^[^ ^50^ ^]^ PTMs perceive different metabolic status resulted from the environment factors (lifestyles, nutrition, and microbes), produce neoantigen presented by the most crucial genetic factors (HLA alleles), and induce immune dysregulations (autoreactive T cell and B cell activations and autoimmunity). Therefore, it is important to attain a more profound grasp of the processes by which PTMs occur and the mechanisms driving their associated immune responses.

## 3 Post‐Translational Modified Neoantigens and Autoreactive Cells

As with somatic mutations, protein PTMs alter the primary structure of amino acid sequences, produce neoantigens, and activate autoreactive immune cells. This series of biological processes triggers abnormal immune responses in autoimmune diseases, resulting in tissue and organ damage. However, in the previous studies, the main perspective of understanding the function of PTMs is their effect on protein functions, rather than the production of neoantigens.

### 3.1 Types of PTMs and Their Protein‐Based Immunological Functions

PTMs are chemical reactions that take place on amino acid side chains or peptide bonds after protein synthesis, thereby increasing the diversity of protein structure and function. As a result, the essential functions of cells, including energy conversion, cell metabolism, and information transmission are disrupted, which can lead to the development of diseases. These modifications, including phosphorylation, glycosylation, ubiquitination, acetylation, and methylation, regulate different cellular processes. In some diseases, metabolic abnormalities arise within the body, and cells sense metabolic changes through the alteration of metabolic induced‐protein PTMs. The metabolic changes include the increase of the tricarboxylic acid (TCA) cycle, oxidative stress damage, lactate accumulation, altered microbial status, etc., can induce changes in metabolites which chemically react with amino acid residues of the protein. Such chemical reactions will lead to the formation of PTMs and may change the original function of proteins. Under different environmental conditions, these PTMs can influence the immune functions of proteins, affect different immunocytes, such as macrophages and T cells, and modulate immune responses and inflammation.^[^ ^51^ ^]^

#### 3.1.1 Alkylation of Cysteine Residues by Itaconate

Itaconate is synthesized from cis‐aconitate via the catalytic action of immune‐responsive gene 1(IRG1).^[^ ^52^ ^]^ Pathogen infections could trigger metabolic reprogramming. Under bacterial infection conditions, LPS‐activated macrophages express IRG1, resulting in increasing the production of itaconate in an IRG1‐dependent manner.^[^ ^53^ ^]^ Cysteine residues in proteins undergo covalent modification through a Michael addition reaction with the unsaturated double bond of itaconate.^[^ ^54^ ^]^ 4‐Octyl Itaconate (4‐OI) which is a derivative of itaconate, alkylates stimulator of interferon genes (STING) by modifying cysteine residue Cys91 and Cys147, restricts cGAS‐STING activation, and suppresses the downstream inflammatory factors.^[^ ^55^ ^]^ In cGAS‐STING‐related autoimmune cell models and murine DNA virus infection models, it is found that 4‐OI has an inhibitory effect on cGAS‐STING.^[^ ^55^, ^56^ ^]^ Mouse treated with ovalbumin and *Chlamydia muridarum* develop infiltration of immune cells and airway inflammation. 4‐OI reduces bronchoalveolar lavage fluid macrophages and airway resistance and inhibits Janus kinase 1 (JAK1) activation in lung tissue.^[^ ^57^ ^]^ The NLRP3 inflammasome mediates the activation of caspase‐1, which facilitates the maturation of IL‐1β, which leads to the development of inflammation.^[^ ^58^ ^]^ 4‐OI modifies Cys548 of NLRP3, potentially explaining the mechanism of the itaconate inhibit inflammation.^[^ ^59^ ^]^

#### 3.1.2 Lactylation of Lysine by Lactate

The tissue microenvironment in cancer and inflammatory diseases exhibits hypoxic metabolism, characterized by elevated levels of lactate and other metabolic byproducts. In the microenvironment of inflammatory diseases represented by rheumatoid arthritis synovium, lactate is produced by infiltrating immune cells and stromal fibroblasts with active metabolism. Meanwhile, various immune cells can detect extracellular lactate levels and modulate their functions through several mechanisms, with lactylation being a crucial process.^[^ ^60^ ^]^ There are various studies have identified a connection between protein lactylation and the pathogenesis of diseases, such as tumors, neurodegenerative disorders, and inflammatory diseases.^[^ ^61^ ^]^

Bacterial infections and hypoxia induce the production of lactate through glycolysis, which leads to histones lactylation including H3K18la. During the later stages of M1 macrophage polarization, the lactylation of histone promotes the transcription and expression of genes involved in wound healing and M2‐like genes.^[^ ^62^ ^]^ The alanyl‐tRNA synthetases AARS1/2 function as sensors for L‐lactate and lysine lactyltransferases.^[^ ^63^ ^]^ AARS2 mediates the lactylation of cGAS at Cys156, which causes it can't sense self‐DNA, which in turn reduces innate immune surveillance and exerts an immunosuppressive function.^[^ ^63b^ ^]^ The protein kinase R‐like ER kinase drives glucose metabolism, resulting in lactate‐driven histone lactylation. In monocyte‐derived macrophages, this process promotes IL‐10 expression, which is important for the suppression of T‐cell activity.^[^ ^64^ ^]^

#### 3.1.3 Succination of Cysteine by Dimethyl Fumarate

Gene mutations, exogenous supplementation, and transformation of cell metabolism to aerobic glycolysis can all increase the intracellular levels of fumarate, thereby affecting immune cells function. The function of fumarate hydratase (FH) is to catalyze the reversible conversion of fumarate to malate. A loss of FH activity is associated with renal cell carcinoma and hereditary leiomyomatosis. The absence of FH leads to significant metabolic alterations, including the accumulation of metabolites such as argininosuccinate and fumarate.^[^ ^65^ ^]^ In tumor cells, FH depletion causes fumarate to accumulate in the tumor microenvironment. The fumarate can succinate ZAP70 at the Cys96 and Cys102 sites, thereby inhibiting its activity in infiltrating CD8^+^ T cells. As a result, the activation of CD8^+^ T cells is diminished, leading to suppressed antitumor immune responses.^[^ ^66^ ^]^

Dimethyl fumarate (DMF) as a drug with immunomodulatory properties, is utilized for treating both psoriasis and MS. DMF has been shown to induce monomethyl and dimethyl (2‐dimethyl succinyl‐cysteine) succination of glyceraldehyde‐3‐phosphate dehydrogenase (GAPDH) at Cys 152 in MS patients undergoing DMF treatment. In activated macrophages and T cells, this modification of GAPDH downregulates aerobic glycolysis, contributing to its anti‐inflammatory properties.^[^ ^67^ ^]^ The pore‐forming protein gasdermin D (GSDMD) is a key factor mediating pyroptosis which contributes to inflammatory disease. Endogenous fumarate or DMF can succinate gasdermin D at Cys191, which limits its ability to initiate cell death by hindering its interaction with caspases. In MS patients’ peripheral blood mononuclear cells (PBMCs), the level of GSDMD‐N and IL‐1β are elevated. With DMF treated, both GSDMD‐N and IL‐1β were reduced in patients.^[^ ^68^ ^]^

#### 3.1.4 Redox Modifications of Cysteine by Reactive Oxygen Species

In many diseases, like systemic inflammatory response syndrome, hypertension, ischemia‐reperfusion injury, and cancer, oxidative stress arises as a secondary effect following the onset of pathology initiated by other factors. The tissue damage leads to the production of the •O^2−^ or H_2_O_2_ from NADPH oxidase and mitochondrial electron transport chain. Redox regulation of cellular functions involves protein redox modifications, where reactive thiols on specific cysteine residues are converted from their reduced form to an oxidized form. The primary types of thiol modification include protein S‐glutathionylation, S‐nitrosylation, and so on.^[^ ^69^ ^]^ Reactive oxygen species (ROS) is believed to lead to the damaged biomolecule accumulation. These damaged and oxidized biomolecules are, in turn, considered to be underlying factors in a range of pathologies, such as neurodegenerative diseases, atherosclerosis, and aging.^[^ ^70^ ^]^

Research has shown that under oxidative stress, S‐glutathionylation of the Cys127 residue in Fatty acid‐binding proteins 5 enhances nuclear translocation and its binding of fatty acid, activating PPARβ/δ and inhibiting macrophage inflammation.^[^ ^71^ ^]^ In human myeloid cells, IFN‐γ stimulates the formation of the IFN‐γ‐activated inhibitor of translation (GAIT) complex, which binds to the GAIT element within the 3′ untranslated regions of various mRNAs related to inflammation and inhibits their translation, like vascular endothelial growth factor (VEGF‐A).^[^ ^72^ ^]^ Upon treatment of cells with oxidatively modified low‐density lipoprotein and IFN‐γ, the S‐nitrosylation of the Cys247 site on GAPDH occurs. This modification prevents GAPDH from interacting with L13a, leading to the proteasomal degradation of L13a. As a result, the GAIT complex becomes dysfunctional, ultimately inducing the expression of the inflammation‐related protein VEGF‐A.^[^ ^73^ ^]^

In addition to immune regulation, common modifications are also widely involved in tumor genesis, infectious diseases, and degenerative diseases (**Table** **1**). Adding phosphate groups to serine, tyrosine, or threonine to control enzyme activity and cell signaling (e.g., p53 phosphorylation) is associated with cancer.^[^ ^74^ ^]^ Glycosylation mutations in the V4 region of HIV envelope protein mediate immune escape partially by affecting antibody binding to the virus.^[^ ^75^ ^]^ Ubiquitination modification can regulate the occurrence and development of tumors, neurodegenerative diseases, autoimmune diseases, and other diseases through multiple pathways. In immunocytes, abnormal ubiquitination can mediate TNFAIP3‐related inflammatory activation,^[^ ^75^ ^]^ protein degradation, etc.^[^ ^76^ ^]^ What's more, acetylation, methylation, and other PTMs act as driving forces of cancer and autoimmune diseases.^[^ ^77^ ^]^ All of the above functions are achieved through protein molecules, and these post‐translational modified proteins have changed the protein activity or interacting partners. Beyond that, citrullination and carboxyethylation, through turning arginine into citrulline or adding a carboxyethyl group to cysteine, change the primary structure of the amino acid sequences, and become the post‐translational modified neoantigens in RA and AS.^[^ ^50^, ^78^ ^]^

**Table 1:** XX XX.

Metabolite related PTM types	Chemical structures	Metabolites	Description	Biological roles	Disease association
**Phosphorylation**	Serine phosphorylation 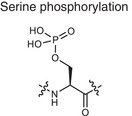	ATP	Adding phosphate groups to proteins	Controls enzyme activity and cell signaling, **Creates PTM neoantigens**	Cancer (e.g., p53 phosphorylation)
**Glycosylation**	Serine O‐GlcNAcylation 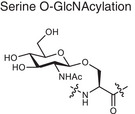	Monosaccharide, glycan	Attaching sugars to proteins	Helps proteins fold and cells communicate **Creates PTM neoantigens**	Infectious diseases (e.g., HIV gp120 glycosylation)
**Acetylation**	Lysine acetylation 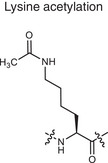	Acetyl‐CoA	Adding acetyl groups to proteins	Affects gene expression and chromatin structure	Cancer (e.g., histone acetylation), autoimmune diseases (e.g., SLE, RA, MS)
**Methylation**	Lysine methylation 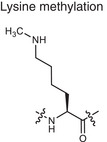	SAM	Adding methyl or dimethyl groups to proteins	Regulates gene expression and protein function **Creates PTM neoantigens**	Cancer (e.g., histone methylation), autoimmune diseases (e.g., SLE, RA, MS)
**Citrullination**	Citrullination 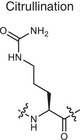	Arginine	Turning arginine into citrulline	**Creates PTM neoantigens** that trigger immune attacks	Autoimmune diseases (e.g., RA)
**Alkylation**	Cysteine alkylation 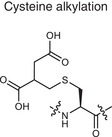	Itaconate	Alkylating the proteins through thia‐Michael addition reaction	Inhibits inflammation and regulates metabolism	Autoimmune diseases (e.g., RA, MS, psoriasis)
**Lactylation**	Lysine lactylation 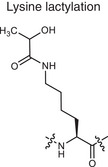	Lactate	Adding a lactyl group to lysine	Regulates gene expression and protein function	Cancer (e.g., histone lactylation), infectious diseases
**Succination**	Cysteine succination 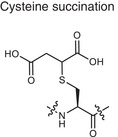	Fumarate	Forming S‐(2‐succinyl) cysteine through a Michael addition reaction	Affects gene expression and protein function	Autoimmune diseases (e.g., MS)
**Carboxyethylation**	Cysteine carboxyethylation 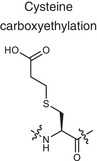	3‐HPA	Adding a carboxyethyl group to proteins	**Creates PTM neoantigens**	Autoimmune diseases (e.g., AS)

Adenosine triphosphate (ATP), S‐adenosyl methionine (SAM), 3‐hydroxypropionic acid(3‐HPA), Systemic lupus erythematosus (SLE), Rheumatoid arthritis (RA), Multiple sclerosis (MS), Ankylosing spondylitis (AS)

### 3.2 Thymic Selection and PTM‐Derived Neoantigens

To better understand and highlight the mechanism of how PTMs break immune tolerance and become pathogenic neoantigens, the main processes of thymus selection (especially the physiology formation of immune tolerance status) should not be neglected.

#### 3.2.1 Thymic Selection

**Figure 2:**
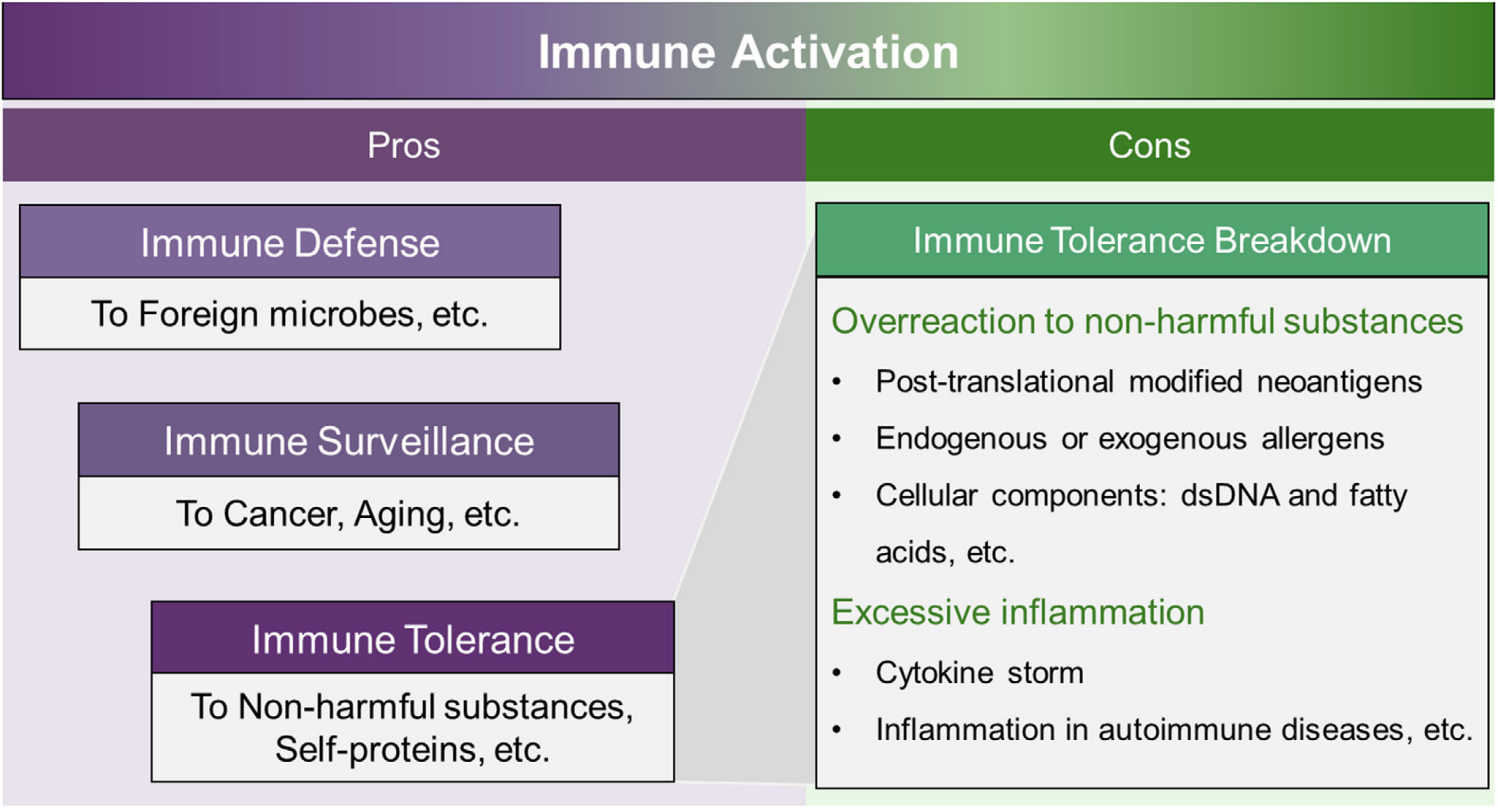
The pros and cons of immune activation. The activation of the immune system plays a vital biological function in the body. The pros of immune activation include immune defense response to foreign pathogenic microorganisms, immune surveillance response to tumors and aging factors, and immune tolerance to non‐harmful substances, self‐proteins and peptides, etc. However, most of the damage caused by immune activation is related to the destruction of immune tolerance, such as overreaction to non‐harmful substances and a persistent inflammatory state in the body.

The power of the immune system lies in distinguishing the non‐self components from self ones.Consequently, the immune system supports key functions such as immune defense, immune surveillance, and immune tolerance (**Figure** **2**). This skill is rooted in the specialized mechanisms of thymus development. To recognize and be tolerant to self‐components, thymic selection is a fundamental mechanism for establishing central tolerance, ensuring that autoreactive T cells are eliminated before they can enter the peripheral immune system.^[^ ^79^ ^]^

Some researchers suppose that neoantigens may trigger immune responses that are initially asymptomatic and then gradually progress into a clinical disease. The interactions between autoreactive T cells and these self‐antigens are prominently featured in autoimmune disorders like myasthenia gravis, MS, and murine EAE. These diseases are usually onset at certain ages, which is mostly after the immune system fully matured. In that case, thymus function is markedly degenerated and immune tolerance almost ceased. Newly appeared antigens including PTM‐derived neoantigens can activate autoreactive T cells. These autoreactive T cells identify specific peptide antigens^[^ ^80^ ^]^ and promote the antigen‐specific cytotoxic effects or antibody responses to induce autoimmunity.

Therefore, the screening of post‐translationally modified protein and peptide profiles, as well as the analysis and identification of modified neoantigens, are very important aspects of the research of the pathogenic mechanism of autoimmune diseases.

#### 3.2.2 PTM‐Derived Neoantigens

PTMs change the primary structure of amino acid sequences, which may lead to the different functions of the protein, as well as generate neoantigens by breaking immune tolerance in autoimmune diseases.

Neoantigens are antigens absent in normal tissues that emerge under pathological conditions, such as those derived from genomic mutations, abnormal transcriptome variants, PTMs, and viral open reading frames (ORFs). There may be limited genomic mutations and ORF‐derived neoantigens in normal tissues based on the pathogenesis of autoimmune diseases. However, abnormal PTMs caused by metabolic disorders could offer an important source of pathogenic PTM‐derived neoantigens. The essence of PTM is to alter the chemical groups of the side chains of the original amino acid residues. PTMs, like mutations at the gene level, can alter the physicochemical properties of the side chains in the original amino acid sequence. Thus, modification groups can be directly involved in the formation of epitopes. Additionally, some epitopes of natural proteins may be hidden due to folding, and PTMs may destroy the original protein structure and expose the hidden epitopes. If the physicochemical changes of peptides do not occur during the immune system development and do not experience negative selection of the thymus, then it is very likely that these peptides will be considered foreign antigens which may induce antigen‐specific immune responses after the immune system matures.

The PTMs may significantly expand the repertoire of ligands for T cell recognition. The influence of PTMs on the physicochemical properties of proteins can modulate the binding affinity to HLA and influence T‐cell responses. There is evidence that peptides containing PTMs, including phosphorylation, cysteinylation, and glycosylation, diversify a library of MHC‐bound peptides, which may be recognized by TCR and subsequently trigger cell activation.^[^ ^81^ ^]^ During processes such as inflammation, cellular transformation, infection, apoptosis, and aging, alterations in the quantity and/or quality of PTMs can modify MHC‐associated peptides. These changes might play an important role in immune responses related to autoimmune disorders^[^ ^81^ ^]^

Based on this logic, it is of great significance to systematically screen the PTM profile of autoimmune patients and find pathogenic PTM forms and PTM antigens for finding the delicate pathogenesis of autoimmune diseases and proposing individualized treatment plans. There are two ways to identify the PTMs of naturally processed MHC‐associated peptides: directly, by the use of mass spectrometry;^[^ ^81^ ^]^ indirectly, the cells presenting the peptide are recognized by T cells that demonstrate specificity for the synthetic modified peptide.

##### Phosphopeptides as Antigens

Protein phosphorylation is a common PTM, where a phosphate group from the ATP or GTP is transferred to serine, threonine, or tyrosine, catalyzed by protein kinases. Changes in protein phosphorylation, such as those involved in cytoplasmic signaling pathways, cell cycle regulation, and metabolism^[^ ^82^ ^]^ may result in the surface presentation of neoantigens derived from phosphopeptides.^[^ ^83^ ^]^

A diverse array of phosphopeptides can be potential antigens.^[^ ^81^, ^84^ ^]^ These phosphorylated peptides have a very conserved motif with a basic arginine or lysine residue in P1 which could enhance the binding affinity of peptides that typically exhibit low affinity.^[^ ^85^ ^]^ Meanwhile, phosphorylation at position P4 might improve immunogenicity either by directly exposing the phosphorylation site to the TCR or by inducing a conformational change in the peptide.^[^ ^84^ ^]^ The difference in the recognition of phosphopeptides and non‐phosphopeptides by T cell receptors may be due to changes in the shape and electrostatic compatibility.

Phosphopeptides are detected in cancerous and healthy tissues, some phosphopeptide epitopes in melanoma and leukemia are cancer‐specific.^[^ ^86^ ^]^ Angela L. Zarling et al. detect over 60 phosphorylated peptides in the Epstein‐Barr virus‐transformed B lymphoblastoid cell line. CD8^+^ T cells can distinctly differentiate between phosphorylated peptides derived from the MUM2 protein and their non‐phosphorylated counterparts. Additionally, cells specific to the phosphorylated peptides are capable of proliferation and growth.^[^ ^87^ ^]^ After that, they identify 36 phosphorylated peptides presented by HLA‐A*02 in B lymphoblastoid cell lines, melanoma cells, and ovarian cancer cells. The phosphorylated IRS2 peptide exhibits a fourfold higher affinity for HLA‐A*02 compared to its non‐phosphorylated counterpart. Additionally, Mouse CD8^+^ T cells specifically recognize the phosphorylated IRS2 peptide presented by dendritic cells and secrete IFN‐γ.^[^ ^82a^ ^]^ Mark Cobbold et al. identify 10 HLA‐A2‐restricted phosphorylated peptides and 85 HLA‐B7‐restricted phosphorylated peptides in leukemia‐associated tumors. T cells isolated from healthy donors demonstrate the ability to specifically recognize the phosphorylated peptides and secrete IFN‐γ in response. These T cells could bind to HLA‐B7‐phosphopeptide tetramers, recognize, and kill tumor cells.^[^ ^88^ ^]^

##### Arginine (di)methylated Peptides as Antigens

Protein arginine methylation is facilitated by protein arginine methyltransferases.^[^ ^89^ ^]^ Arginine methylation occurs in three forms: monomethylated, symmetric, or asymmetric dimethylated arginine residues. The role of arginine methylation has been explored concerning gene expression regulation and DNA repair mechanisms. Additionally, protein arginine methylation is associated with pathogenic processes such as cancer development, autoimmune disorders, and viral infections.^[^ ^90^ ^]^

Yagüe et al. first report a natural HLA‐B*39 ligand carrying a dimethylated Arg residue from an RNA‐binding nucleoprotein. This dimethylated arginine is centrally positioned in the peptide, potentially presenting the dimethyl‐Arg on the HLA‐peptide complex surface for T‐cell receptor interaction.^[^ ^91^ ^]^ Subsequently, Saulius Jarmalavicius et al. identify the methylation peptide derived from the regulator of the nuclear corepressor GPS‐2.^[^ ^92^ ^]^ The immune system specifically recognizes the methylation status, as only the monomethylated variant induces T cell response, and this response is significantly stronger in patients compared to healthy controls.^[^ ^92^ ^]^ Fabio Marino et al. describe 149 arginine (di)methylated peptides restricted to HLA class I. Among these methylated peptides bound to HLA‐B*07 predominantly feature dimethylation at the P3 position.^[^ ^89^, ^92^ ^]^

##### O‐GlcNAc–Modified Peptides as Antigens

O‐linked β‐N‐acetylglucosamine (O‐GlcNAc) is a PTM that adds a single β‐D‐N‐acetylglucosamine (GlcNAc) unit to the threonine or serine residues of proteins.^[^ ^93^ ^]^ O‐GlcNAc modifications occur on a variety of proteins, such as transcription factors, cytoskeletal proteins, signal molecules, and kinases. Furthermore, O‐GlcNAc modifications are extensively involved in diverse biological processes, such as metabolic regulation, transcriptional regulation, cell growth, maintenance, and signal transduction.^[^ ^94^ ^]^ O‐GlcNAcylation is influenced by a variety of upstream factors, such as insulin signaling, glucose metabolism, and cellular stress response.^[^ ^95^ ^]^ Malaker et al. identify 36 peptides modified by O‐GlcNAc that are presented by the HLA‐B*07 molecule from leukemia samples. The GlcNAc residue is positioned centrally within the peptide, possibly optimizing it for T cell recognition. O‐GlcNAcylated peptides elicit robust cytotoxic phenotypes and memory T cell activation in healthy donors.^[^ ^96^ ^]^

##### Citrullinated Peptides as Antigens

Citrullination, catalyzed by peptidyl arginine deiminase, involves the hydrolysis of positively charged arginine into neutral citrulline and urea. Abnormal protein citrullination is associated with diseases like RA, SLE, and cancer.^[^ ^97^ ^]^ Anti‐cyclic citrullinated peptide antibodies are commonly found in the blood of most RA patients, making it a useful tool for auxiliary diagnosis.^[^ ^98^ ^]^ Citrullinated peptides derived from proteins such as fibrinogen‐α and actin have been identified in the synovium. Among these, RA sera specifically exhibit reactivity to the selected citrullinated vimentin epitope.^[^ ^99^ ^]^ In addition to HLA‐DR presenting citrullinated self‐antigens to T cells, citrullination can alter the self‐antigens processing and presentation. This alteration facilitates the creation and display of unique cryptic epitopes, which can activate autoreactive T cells in individuals with RA.^[^ ^100^ ^]^

##### Cysteine Carboxyethylation Peptide as Antigen

Protein carboxyethylation is catalyzed by the CBS enzyme, where 3‐hydroxypropionate (3‐HPA) reacts with cysteine thiol groups to form S‐(1‐carboxyethyl)‐Cys. In patients with AS, elevated plasma levels of 3‐HPA induce carboxyethylation at the Cys96 residue of ITGA2B. This post‐translationally modified ITGA2B undergoes lysosomal degradation, generating antigenic peptides ITGA2B‐ceC96 that can be presented by HLA molecules. Specifically, HLA‐DRB1*04 presents this neoantigen to CD4^+^ T cells, triggering an immune response and inducing the production of antibodies against the ITGA2B‐ceC96 antigenic peptide in AS patients.^[^ ^50a^ ^]^ T cell and B cell autoreactivity to modified peptides induce inflammation and damage, leading to the pathogenesis of AS in mice.^[^ ^101^ ^]^

**Figure 3:**
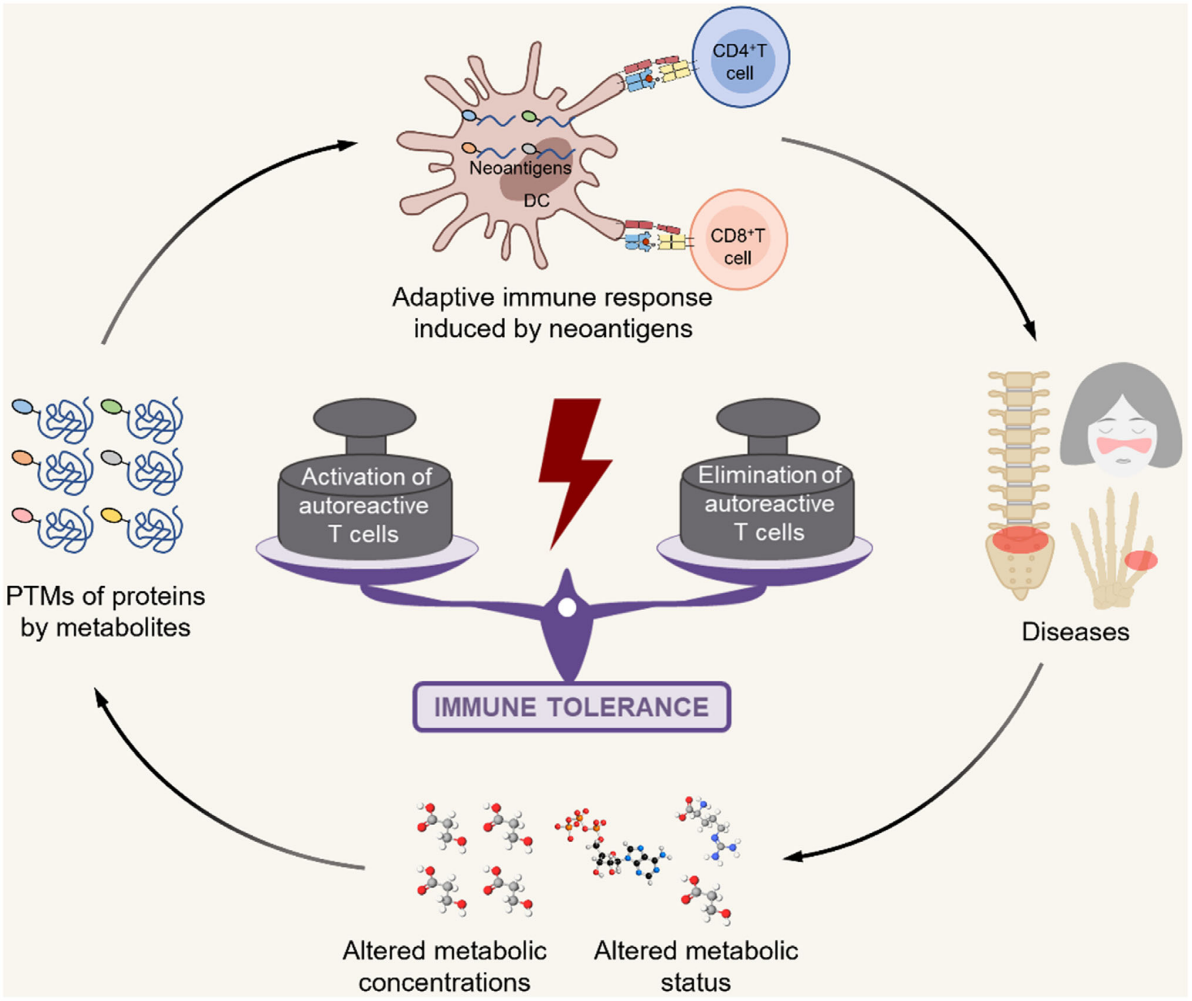
PTMs resulted from metabolic change produce neoantigens, activate self‐reactive T cells, and trigger autoimmune diseases. The status of metabolic conditions and the concentration of metabolites change in disease. These metabolites subsequently lead to PTMs of proteins. The neoantigens derived from these modifications are presented by antigen‐presenting cells, activating self‐reactive T cells, breaking immune tolerance, and thereby triggering or exacerbating diseases, such as autoimmune disease.

Taken together, these post‐translationally modified peptides all have antigenic properties and are nascent epitopes. However, in order to cause tissue and organ damage, in addition to the fact that the peptide does not appear during the processes of thymus selection and can be presented by MHC molecules, it is also necessary to activate specific T cells to induce the expansion and activation of such autoreactive T cells (**Figure** **3**).

## 4 Diagnostic and Therapeutic Potential of PTM‐Derived Neoantigens

Although many autoimmune diseases have well‐established diagnostic methods, some autoimmune diseases, such as AS, are often diagnosed with irreversible tissue and organ damage, such as sacroiliac joint ankylosis. Interventions based on this stage of the disease are very limited in reversing and arresting the advancement of the disease. The post‐translational modification‐based diagnosis and therapeutics will enable the development of individualized treatment for patients in the early phases of the disease.

### 4.1 PTM‐Derived Neoantigens‐Based Biomarkers for Autoimmune Disease Diagnosis

Post‐translationally modified neoantigens can be used as biomarkers in the diagnosis of autoimmune diseases. Moreover, such biomarkers are not limited to neoantigens, but also related metabolic substrates, HLA genotyping, PTM levels, autoreactive T cell amplification, and autoantibody levels can play an important diagnostic and monitoring role in all stages of the disease. In RA, citrullinated peptides derived from proteins like vimentin and fibrinogen can act as disease‐specific biomarkers. Research has shown that anti‐citrullinated protein antibodies (ACPAs) are present in ≈70% of RA patients, making them a critical diagnostic tool.^[^ ^102^ ^]^ Likewise, another neoantigen‐specific autoantibody assessment research has proved that anti‐cysteine carboxyethylated autoantibody is associated with AS.^[^ ^50b^ ^]^ Similarly, in SLE, PTM‐derived neoantigens such as acetylated histones have been implicated in disease pathogenesis, with studies demonstrating their presence in patient sera and their correlation with disease activity.^[^ ^103^ ^]^

In contrast, modified metabolic substrate detection and neoantigen‐specific autoreactive T‐cell detection are underestimated. It's found that HLA‐DR^+^ β1‐integrin^+^ CD8^+^ T cells present in the peripheral circulation of pediatric patients diagnosed with UC, in which positively correlate with systemic and mucosal inflammation biomarkers.^[^ ^46^ ^]^ Given the challenges in achieving a precise diagnosis, such lymphocytes may serve as a valuable additional diagnostic instrument.^[^ ^104^ ^]^ On the other hand, as there exist TCR repertoires and clonotypes specific for T1D, the integration of extensive TCR sequence data originated from T1D patients and healthy controls, combined with advanced bioinformatics analysis will aid in developing TCRs as potent biomarkers for T1D progression.^[^ ^105^ ^]^

A more refined and promising strategy contains two parts: the assessment of disease risk and disease activity. First, the assays for metabolites, PTM‐derived neoantigen, and HLA haplotype, this series of assessment can detect pathogenicity associated with PTM‐derived neoantigens in the initial phase and can be used to assess the risk of disease at a very early stage in individuals who are susceptible to autoimmune diseases. And then, the production of autoreactive lymphocytes and autoantibodies could also be tested to verify the disease activity based on the disease‐related PTM‐derived neoantigens. With the discovery and identification of broader PTM‐derived neoantigen profiles, this evaluation system based on disease‐related PTM‐derived neoantigen will become more feasible and have more diagnosis and treatment value for autoimmune patients.^[^ ^106^ ^]^

### 4.2 Potential Therapeutic Tips Targeting Neoantigens Induced Autoreactive T Cells

**Figure 4:**
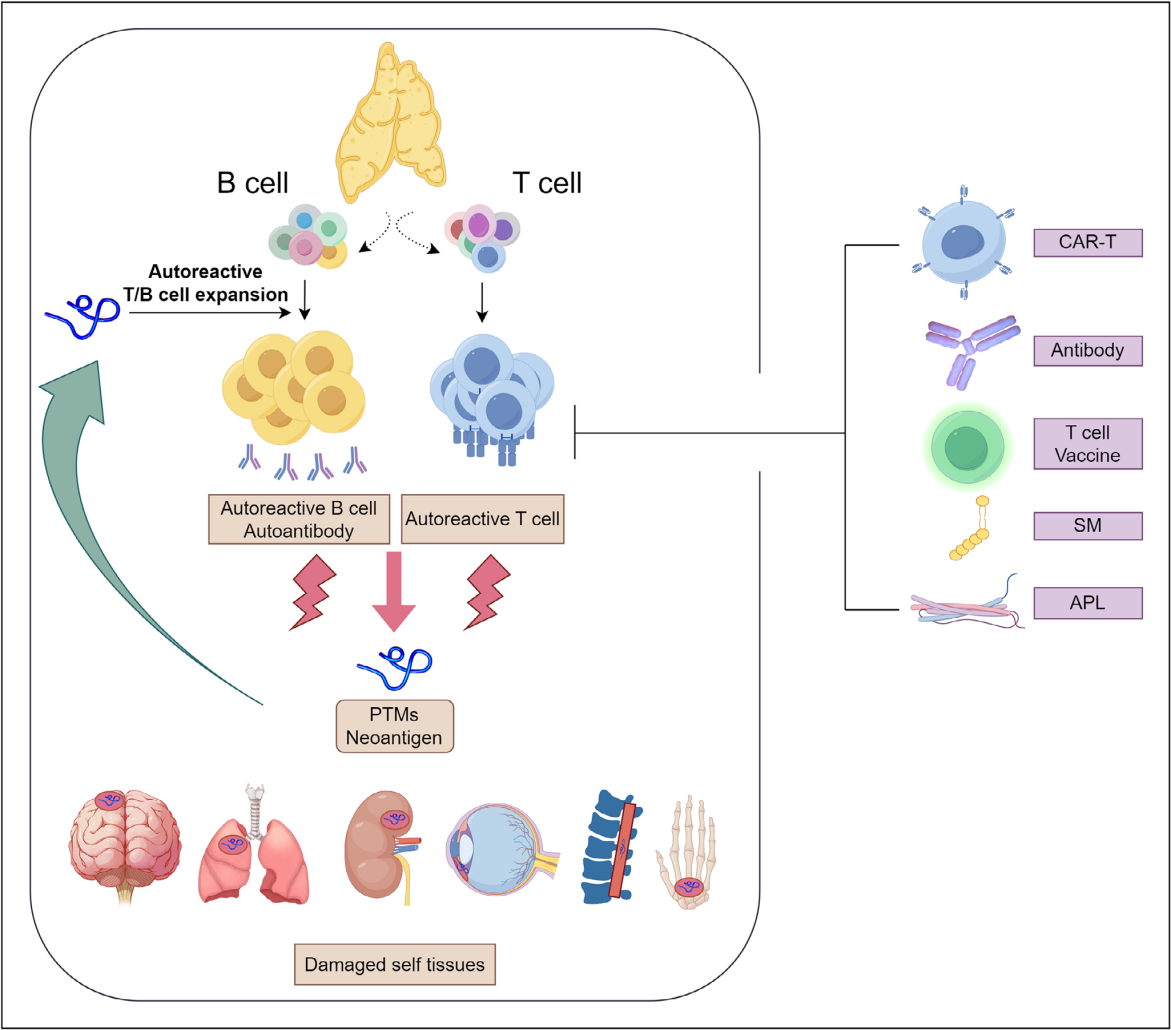
The overview of how autoreactive cells generate, take effect, and their targeted elimination. Incomplete clonal deletion and the presence of neoantigen may cause the activation and expansion of autoreactive T and B cells that would recognize and impair self‐tissues, eventually resulting in autoimmune disorders. There are several therapies including anti‐T cell antibodies, peptide‐MHC CART, T cell‐based vaccination, aimed to suppress and delete target autoreactive T cells so as to suppress and delete them, and finally interfere with the autoimmunity process and treat autoimmune diseases. By Figdraw (http://www.figdraw.com).

As for PTM or neoantigen, there have been some attempts to target them in cancer therapies, including tumor vaccines, adoptive cell therapy, and antibody therapies, as well as potential predictors for immune checkpoint blockades.^[^ ^107^ ^]^ However, in autoimmune diseases, the newly appeared PTMs resented on the normal tissue surfaces are usually harmless without abnormal autoimmunity responses, and therapies targeting PTM sites may not be effective. The key reason is that pathogenicity does not arise from post‐translationally modified neoantigens, but from aberrantly activated self‐reactive lymphocytes. That is, when the post‐translationally modified neoantigens are targeted, such “treatment” may mimic the process of autoimmunity and thus exacerbate the disease (**Figure** **4**). The promising ways of disease treatment is to induce apoptosis of autoreactive lymphocytes which references to the auto‐tolerant process of the thymus during the development of the immune system. Thus, several therapies for the downstream key effector autoreactive T cells seem promising.

#### 4.2.1 Chimeric Antigen Receptor‐T Cell Immunotherapy

The principle of CAR‐T therapy lies in precisely killing diseased cells with the high specificity of an antibody by the strong power of T cell. It has greatly changed the field of oncology, autoimmune diseases, cardiac disease, senescence‐associated diseases, and other diseases.^[^ ^108^ ^]^ However, to specifically recognize and kill autoreactive T cells, the construct of recognizing part may not be the antibody for now. There have been some researches attempting to target the TCR of autoreactive T cells since 2002,^[^ ^109^ ^]^ which can specifically eliminate key pathogenic T cells in the disease immune environment, with high specificity and low toxicity. CAR‐T with peptide‐MHC as the recognition area and targeting the TCR of autoreactive T cells has achieved models and obtained better experimental results in experimental autoimmune encephalomyelitis,^[^ ^110^ ^]^ type 1 diabetes,^[^ ^111^ ^]^ and other diseases. However, the way to construct modified peptide‐MHC in CAR is still a huge challenge.

#### 4.2.2 Antibodies Targeting Autoreactive T Cell

Antibody therapy has been widely applied in many kinds of diseases and achieved great advancement. Likewise, antibodies targeting autoreactive T cells, particularly TCR, is also an essential idea for neoantigen‐based disease treatment. Since 1987, studies in mice have investigated antibodies targeting Vβ regions commonly found on alloreactive T cells, revealing that mice with this specific alloantigen exhibited 1‐20‐fold reduction in the number of T cells expressing such Vβ gene.^[^ ^112^ ^]^ Inducing exhaustion of autoreactive T cells reflects potential as a therapeutic intervention for SLE.^[^ ^113^ ^]^ Early initiation of anti‐CD4 monoclonal antibody therapy following hematopoietic cell transplantation facilitates thymus regeneration. This strategy aids in minimizing damage to thymic epithelial cells, sustains thymic selection, and concurrently preserves the graft‐versus‐leukemia response.^[^ ^114^ ^]^ Additionally, it is reported that treatment with anti‐TRBV9 antibodies may result in the elimination of human TRBV9^+^ T cells in an AS patient, who achieved remission in three months and has been sustained for four years.^[^ ^115^ ^]^

#### 4.2.3 Neoantigen Vaccination and T Cell‐Based Vaccination

The citrullinated self‐antigen vaccine, citAg, has demonstrated strong immunogenicity, capable of stimulating PBMCs from RA patients to secrete IL‐6 and IFN‐γ. The citAg vaccine can induce immune tolerance through mechanisms such as energy and exhaustion, leading to reduced frequencies of Th17 and Th1 cells and antigen‐specific T cell responses, thereby improving experimental arthritis. This provides a novel therapeutic strategy for RA.^[^ ^116^ ^]^ On the other hand, T cell‐based vaccination (TCV) also holds promise as a preventive measure for autoimmune diseases. In 1981, it was first proposed in an EAE rat model using MBP‐reactive T‐cells which are activated and then irradiated as vaccines.^[^ ^117^ ^]^ Multiple clinical trials of TCV have been conducted across various phases of MS. Among them, a double‐blind, sham‐controlled trial in patients with relapsing‐progressive MS utilizing anti‐myelin cell lines has illustrated the procedure's feasibility and safety, while also offering preliminary evidence of TCV's clinical efficacy in MS.^[^ ^118^ ^]^ Hence, the protective immunity generated by either moderated or pre‐manipulated autoreactive cells continued to be advantageous in the future.

#### 4.2.4 Sphingomyelin for CD1 Autoreactive T Cells

Except metabolic‐related PTMs could produce neoantigen for vaccination, there are also some other metabolites which can directly act as neoantigens and be targeted to block the interaction between antigen and immune cells. CD1a autoreactive T cells activated by lipid presentation are overrepresented in human skin lesions associated with atopic dermatitis and psoriasis, and the component of C42, doubly unsaturated sphingomyelin (42:2 SM) may function as an inhibitory agent by obstructing the possible binding of TCRs presented on CD1a self‐reactive T cells.^[^ ^119^ ^]^ During stable physiological conditions, the preferential loading of “blocker” lipids into CD1a disrupts TCR recognition. Adjusting the lipids loaded into CD1a could potentially serve as a therapeutic strategy to control this autoreactivity.^[^ ^120^ ^]^

#### 4.2.5 Altered Peptide Ligands

Altered peptide ligands (APLs) are another therapeutic peptides generated by the structural modification of single conservative amino acid substitutions in antigen‐derived immunodominant peptides,^[^ ^121^ ^]^ similar to PTMs neoantigens which are also called “naturally occurring APLs”. However, contrary to the pathogenic effect of neoantigen, APLs can effectively suppress expansion without affecting cytokine and antibody production. Through enhancing T‐B cell collaboration and stimulating the development of Tregs rather than Th cell polarization, or acting as super agonists, these APLs achieve this effect.^[^ ^122^ ^]^ The development of APLs presents promising and innovative therapeutic opportunities for RA. Initial in vitro screenings have identified APLs with partial agonist effects. These modified peptides can modulate immune responses without triggering unwanted T cell activation. Remarkably, in combination with PBMCs from patients diagnosed with RA, APL‐1, an altered peptide ligand originated from human heat‐shock protein 60 with the D19 mutation to L19, stimulates programmed cell death of autoreactive CD4^+^ CD25^+^ T cells and enhances IL‐10 production.^[^ ^123^ ^]^ The potential of APLs to halt disease progression is evaluated using animal studies. This effect is brought about either directly by shifting the immune response from Th1/Th17 to Th2 or indirectly by the passive administration of T cells that have undergone training by APCs.^[^ ^43^, ^124^ ^]^ Although there are not APLs directly transformed by PTM‐derived neoantigens, we believe that one day, there would be such APLs to conunteract the break of immune tolerance based on how normal peptides are turned into neoantigens and figure out on which position to achieve this.

## 5 Future Research Directions

In the past few decades, researchers have been trying to explore the mechanism of immune tolerance impairment in autoimmune diseases and the search for key antigens from different perspectives. However, it has to be said that the systematic screening of PTM‐derived neoantigens in the true sense has only really begun. This is largely based on advances in modern technologies such as mass spectrometry (MS), which have made it possible to screen for post‐translational modified neoantigens on a large scale.

### 5.1 High‐Throughput Technologies for PTM Identification

High‐throughput technologies have revolutionized the identification of PTMs, enabling large‐scale analysis critical for understanding autoimmune diseases. Mass spectrometry and proteomics lead these advancements, offering precision in detecting PTMs. Recent work used liquid chromatography‐tandem mass spectrometry (LC‐MS/MS) to profile cysteine carboxyethylation in AS patients, identifying 643 unique delta mass clusters across 21716 protein positions.^[^ ^50a^ ^]^ This revealed a significant increase in the 72.021 Da mass shift, corresponding to cysteine carboxyethylation, which provided high‐throughput technologies in uncovering disease‐specific PTMs that may act as neoantigens driving autoimmune responses.

### 5.2 Computational Tools for Predicting PTM‐Derived Neoantigens

Beyond identifying PTMs, computational tools also excel in predicting the immunogenicity of neoantigens. Widely used algorithms like NetMHC and NetMHCpan are instrumental in estimating the binding affinity of neoantigens to MHC molecules, a crucial step in determining their potential to trigger immune responses. More websites and software for predicting antigenicity are also emerging. However, a key problem here is that it is not possible to make effective predictions of forms other than known amino acids, such as post‐translationally modified amino acids. Therefore, it is also hoped that on the basis of powerful computing analysis platforms such as Alphafold, more flexible predictions of PTM‐specific antigens can be developed, which will provide important computational support for subsequent screening and validation, and greatly reduce the investment and waste in experiments.

The comprehensive and precise identification of PTM‐derived neoantigens and their homologous TCR is critical for diagnosis and the treatment of autoimmune diseases. Despite the high throughput technologies and computational tools for screening PTM‐derived neoantigens are getting faster, it is still difficult to accurately identify PTM‐derived neoantigens that induce the autoreactive T cells in patients. Neoantigen vaccines prepared from mutant neoantigens screened in tumors have demonstrated strong immunogenicity and efficacy in targeting tumor cell killing. But how to target the elimination of autoreactive T cells that recognize PTM‐derived neoantigens in autoimmune diseases remains unclear. With an in‐depth understanding of the mechanism of PTM‐derived neoantigens in autoimmune diseases and the precise screening of neoantigens, the ultimate goal is to develop new therapeutic methods for autoimmune diseases, and it is expected that future research can further reduce the clinical symptoms of patients with autoimmune diseases, and even achieve the cure of diseases.

## Conflict of Interest

The authors declare no conflict of interest.

## References

[1] a) G. Rizzuto , J. F. Brooks , S. T. Tuomivaara , T. I. McIntyre , S. Ma , D. Rideaux , J. Zikherman , S. J. Fisher , A. Erlebacher , Nature 2022, 603, 497; 35236989 10.1038/s41586-022-04471-0 PMC9592526

[2] K. Higashioka , D. A. Rao , Immunity 2024, 57, 2715. 39662087 10.1016/j.immuni.2024.11.012

[3] D. Didona , G. Di Zenzo , Front. Immunol. 2018, 9, 779. 29719538 10.3389/fimmu.2018.00779 PMC5913575

[4] C. Fahlquist‐Hagert , T. R. Wittenborn , E. Terczynska‐Dyla , K. S. Kastberg , E. Yang , A. N. Rallistan , Q. R. Markett , G. Winther , S. Fonager , L. F. Voss , M. K. Pedersen , N. van Campen , A. Ferapontov , L. Jensen , J. Huang , J. D. Nieland , C. E. van der Poel , J. Palmfeldt , M. C. Carroll , P. J. Utz , Y. Luo , L. Lin , S. E. Degn , Nat. Commun. 2023, 14, 6941. 37907556 10.1038/s41467-023-42541-7 PMC10618542

[5] S. E. Degn , C. E. van der Poel , D. J. Firl , B. Ayoglu , F. A. Al Qureshah , G. Bajic , L. Mesin , C. A. Reynaud , J. C. Weill , P. J. Utz , G. D. Victora , M. C. Carroll , Cell 2017, 170, 913. 28841417 10.1016/j.cell.2017.07.026 PMC5784431

[6] a) F. W. Miller , Curr. Opin. Immunol. 2023, 80, 102266; 36446151 10.1016/j.coi.2022.102266 PMC9918670

[7] a) H. Benham , H. J. Nel , S. C. Law , A. M. Mehdi , S. Street , N. Ramnoruth , H. Pahau , B. T. Lee , J. Ng , M. E. G. Brunck , C. Hyde , L. A. Trouw , N. L. Dudek , A. W. Purcell , B. J. O'Sullivan , J. E. Connolly , S. K. Paul , K.‐A. Lê Cao , R. Thomas , Sci. Transl. Med. 2015, 7, 290ra87. 10.1126/scitranslmed.aaa9301 26041704

[8] P. Bowness , Annu. Rev. Immunol. 2015, 33, 29. 25861975 10.1146/annurev-immunol-032414-112110

[9] F. Ruzzon , G. Adami , Clin. Exp. Rheumatol. 2024, 42, 1343. 38829018 10.55563/clinexprheumatol/z7lkua

[10] R. Bilski , P. Kamiński , D. Kupczyk , S. Jeka , J. Baszyński , H. Tkaczenko , N. Kurhaluk , Int. J. Mol. Sci. 2024, 25, 7814. 39063056 10.3390/ijms25147814 PMC11277374

[11] a) D. Wang , R. Li , Y. Jin , X. Shen , A. Zhuang , Int. Journal Rheumatic Diseases 2023, 26, 2470; 10.1111/1756-185X.14938 37875269

[12] a) J. M. Hazes , B. A. Dijkmans , J. P. Vandenbroucke , R. R. de Vries , A. Cats , Ann. Rheum. Dis. 1990, 49, 980; 2270970 10.1136/ard.49.12.980 PMC1004291

[13] C. Mölzer , Y. H. Liu , E. Muckersie , I. P. Klaska , R. Cornall , H. M. Wilson , L. Kuffová , J. V. Forrester , Sci. Rep. 2023, 13, 1256. 36690619 10.1038/s41598-022-27018-9 PMC9870966

[14] I. A. Ishina , M. Y. Zakharova , I. N. Kurbatskaia , A. E. Mamedov , A. A. Belogurov Jr. , A. G. Gabibov , Cells 2023, 12, 314. 36672249 10.3390/cells12020314 PMC9856717

[15] H. Larsen , B. Muz , T. L. Khong , M. Feldmann , E. M. Paleolog , Arthritis Res. Ther. 2012, 14, R180. 22866899 10.1186/ar3934 PMC3580575

[16] X. Hu , L. B. Ivashkiv , Immunity 2009, 31, 539. 19833085 10.1016/j.immuni.2009.09.002 PMC2774226

[17] P. Pisitkun , H. L. Ha , H. Wang , E. Claudio , C. C. Tivy , H. Zhou , T. N. Mayadas , G. G. Illei , U. Siebenlist , Immunity 2012, 37, 1104. 23123062 10.1016/j.immuni.2012.08.014 PMC3594848

[18] G. A. M. Povoleri , L. E. Durham , E. H. Gray , S. Lalnunhlimi , S. Kannambath , M. J. Pitcher , P. Dhami , T. Leeuw , S. E. Ryan , K. J. A. Steel , B. W. Kirkham , L. S. Taams , Cell Rep. 2023, 42, 112514. 37195862 10.1016/j.celrep.2023.112514 PMC10790246

[19] S. Culina , A. I. Lalanne , G. Afonso , K. Cerosaletti , S. Pinto , G. Sebastiani , K. Kuranda , L. Nigi , A. Eugster , T. Østerbye , A. Maugein , J. E. McLaren , K. Ladell , E. Larger , J.‐P. Beressi , A. Lissina , V. Appay , H. W. Davidson , S. Buus , D. A. Price , M. Kuhn , E. Bonifacio , M. Battaglia , S. Caillat‐Zucman , F. Dotta , R. Scharfmann , B. Kyewski , R. Mallone , J.‐C. Carel , N. Tubiana‐Rufi , et al., Sci. Immunol. 2018, 3, aao4013. 10.1126/sciimmunol.aao4013 PMC5874133 29429978

[20] a) N. Bao , B. Fu , X. Zhong , S. Jia , Z. Ren , H. Wang , W. Wang , H. Shi , J. Li , F. Ge , Q. Chang , Y. Gong , W. Liu , F. Qiu , S. Xu , T. Li , Int. Immunopharmacol. 2023, 121, 110530; 37348231 10.1016/j.intimp.2023.110530

[21] a) J. A. Encinas , M. B. Lees , R. A. Sobel , C. Symonowicz , H. L. Weiner , C. E. Seidman , J. G. Seidman , V. K. Kuchroo , Int. Immunol. 2001, 13, 257; 11222494 10.1093/intimm/13.3.257

[22] Y. Jing , Y. Kong , J. McGinty , G. Blahnik‐Fagan , T. Lee , S. Orozco‐Figueroa , M. L. Bettini , E. A. James , M. Bettini , Diabetes 2022, 71, 1012. 35179565 10.2337/db21-0993 PMC9044133

[23] a) R. J. Huebner , M. J. Casey , R. M. Chanock , K. Schell , Proc. Natl. Acad. Sci. USA 1965, 54, 381; 4285931 10.1073/pnas.54.2.381 PMC219674

[24] N. McGranahan , A. J. S. Furness , R. Rosenthal , S. Ramskov , R. Lyngaa , S. K. Saini , M. Jamal‐Hanjani , G. A. Wilson , N. J. Birkbak , C. T. Hiley , T. B. K. Watkins , S. Shafi , N. Murugaesu , R. Mitter , A. U. Akarca , J. Linares , T. Marafioti , J. Y. Henry , E. M. Van Allen , D. Miao , B. Schilling , D. Schadendorf , L. A. Garraway , V. Makarov , N. A. Rizvi , A. Snyder , M. D. Hellmann , T. Merghoub , J. D. Wolchok , S. A. Shukla , et al., Science 2016, 351, 1463. 26940869 10.1126/science.aaf1490 PMC4984254

[25] K. He , T. Wan , D. Wang , J. Hu , T. Zhou , W. Tao , Z. Wei , Q. Lu , R. Zhou , Z. Tian , R. A. Flavell , S. Zhu , Cell 2023, 186, 3033. 37327784 10.1016/j.cell.2023.05.027

[26] P. Malekzadeh , A. Pasetto , P. F. Robbins , M. R. Parkhurst , B. C. Paria , L. Jia , J. J. Gartner , V. Hill , Z. Yu , N. P. Restifo , A. Sachs , E. Tran , W. Lo , R. P. T. Somerville , S. A. Rosenberg , D. C. Deniger , J. Clin. Invest. 2019, 129, 1109. 30714987 10.1172/JCI123791 PMC6391139

[27] S. S. Naz , A. Aslam , T. Malik , Infect. Disorders–Drug Targets 2021, 21, 300821192322. 10.2174/1871526521666210317161329 33739247

[28] F. Zhang , K. Wei , K. Slowikowski , C. Y. Fonseka , D. A. Rao , S. Kelly , S. M. Goodman , D. Tabechian , L. B. Hughes , K. Salomon‐Escoto , G. F. M. Watts , A. H. Jonsson , J. Rangel‐Moreno , N. Meednu , C. Rozo , W. Apruzzese , T. M. Eisenhaure , D. J. Lieb , D. L. Boyle , A. M. Mandelin , B. F. Boyce , E. DiCarlo , E. M. Gravallese , P. K. Gregersen , L. Moreland , G. S. Firestein , N. Hacohen , C. Nusbaum , J. A. Lederer , H. Perlman , et al., Nat. Immunol. 2019, 20, 928. 31061532 10.1038/s41590-019-0378-1 PMC6602051

[29] A. M. M. Miltenburg , J. M. Van Laar , R. De Kuiper , M. R. Daha , F. C. Breedveld , Scandinavian J. Immunol. 2006, 35, 603. 10.1111/j.1365-3083.1992.tb03260.x 1349769

[30] T. Kobezda , S. Ghassemi‐Nejad , K. Mikecz , T. T. Glant , Z. Szekanecz , Nat. Rev. Rheumatol. 2014, 10, 160. 24394350 10.1038/nrrheum.2013.205 PMC3953227

[31] A. Hanyecz , T. Bárdos , S. E. Berlo , E. Buzás , A. B. Nesterovitch , K. Mikecz , T. T. Glant , Arthritis Rheumat. 2003, 48, 2959. 14558103 10.1002/art.11275

[32] a) R. Liu , S. Du , L. Zhao , S. Jain , K. Sahay , A. Rizvanov , V. Lezhnyova , T. Khaibullin , E. Martynova , S. Khaiboullina , M. Baranwal , Front. Immunol. 2022, 13, 996469. 36211343 10.3389/fimmu.2022.996469 PMC9539795

[33] I. Bartholomaus , N. Kawakami , F. Odoardi , C. Schlager , D. Miljkovic , J. W. Ellwart , W. E. Klinkert , C. Flugel‐Koch , T. B. Issekutz , H. Wekerle , A. Flugel , Nature 2009, 462, 94. 19829296 10.1038/nature08478

[34] C. H. Goldschmidt , L. H. Hua , Degener Neurol. Neuromuscul. Dis. 2020, 10, 29. 32617031 10.2147/DNND.S224912 PMC7326221

[35] B. J. Kaskow , C. Baecher‐Allan , Cold Spring Harb. Perspect. Med. 2018, 8, a029025. 29358315 10.1101/cshperspect.a029025 PMC5880159

[36] H. Li , A. Boulougoura , Y. Endo , G. C. Tsokos , J. Autoimmun. 2022, 132, 102870. 35872102 10.1016/j.jaut.2022.102870

[37] X. Valencia , C. Yarboro , G. Illei , P. E. Lipsky , J. Immunol. 2007, 178, 2579. 17277168 10.4049/jimmunol.178.4.2579

[38] J. Marks‐Konczalik , S. Dubois , J. M. Losi , H. Sabzevari , N. Yamada , L. Feigenbaum , T. A. Waldmann , Y. Tagaya , Proc. Natl. Acad. Sci. USA 2000, 97, 11445. 11016962 10.1073/pnas.200363097 PMC17219

[39] V. C. Kyttaris , Y. T. Juang , K. Tenbrock , A. Weinstein , G. C. Tsokos , J. Immunol. 2004, 173, 3557. 15322221 10.4049/jimmunol.173.5.3557

[40] D. L. Faustman , M. Davis , J. Mol. Med. (Berl) 2009, 87, 1173. 19693476 10.1007/s00109-009-0516-6

[41] P. S. Apaolaza , D. Balcacean , J. Zapardiel‐Gonzalo , T. Rodriguez‐Calvo , Diabetologia 2023, 66, 1129. 36884056 10.1007/s00125-023-05888-6 PMC10163126

[42] a) K. M. Gillespie , CMAJ 2006, 175, 165; 16847277 10.1503/cmaj.060244 PMC1489998

[43] G. R. Martini , E. Tikhonova , E. Rosati , M. B. DeCelie , L. K. Sievers , F. Tran , M. Lessing , A. Bergfeld , S. Hinz , S. Nikolaus , J. Kumpers , A. Matysiak , P. Hofmann , C. Saggau , S. Schneiders , A. K. Kamps , G. Jacobs , W. Lieb , J. Maul , B. Siegmund , B. Seegers , H. Hinrichsen , H. H. Oberg , D. Wesch , S. Bereswill , M. M. Heimesaat , J. Rupp , O. Kniemeyer , A. A. Brakhage , S. Brunke , et al., Nat. Med. 2023, 29, 2602. 37749331 10.1038/s41591-023-02556-5 PMC10579100

[44] Y. Liu , Q. Lan , L. Lu , M. Chen , Z. Xia , J. Ma , J. Wang , H. Fan , Y. Shen , B. Ryffel , D. Brand , F. Quismorio , Z. Liu , D. A. Horwitz , A. Xu , S. G. Zheng , J. Mol. Cell Biol. 2014, 6, 81. 23861553 10.1093/jmcb/mjt026 PMC3927769

[45] H. Bottois , M. Ngollo , N. Hammoudi , T. Courau , J. Bonnereau , V. Chardiny , C. Grand , B. Gergaud , M. Allez , L. L. Bourhis , Front. Immunol. 2020, 11, 896. 32477365 10.3389/fimmu.2020.00896 PMC7235448

[46] H. Rabe , M. Malmquist , C. Barkman , S. Ostman , I. Gjertsson , R. Saalman , A. E. Wold , Clin. Exp. Immunol. 2019, 197, 111. 30883691 10.1111/cei.13294 PMC6591150

[47] R. Di Niro , L. Mesin , N. Y. Zheng , J. Stamnaes , M. Morrissey , J. H. Lee , M. Huang , R. Iversen , M. F. du Pre , S. W. Qiao , K. E. Lundin , P. C. Wilson , L. M. Sollid , Nat. Med. 2012, 18, 441. 22366952 10.1038/nm.2656 PMC4533878

[48] A. Christophersen , E. G. Lund , O. Snir , E. Sola , C. Kanduri , S. Dahal‐Koirala , S. Zuhlke , O. Molberg , P. J. Utz , M. Rohani‐Pichavant , J. F. Simard , C. L. Dekker , K. E. A. Lundin , L. M. Sollid , M. M. Davis , Nat. Med. 2019, 25, 734. 30911136 10.1038/s41591-019-0403-9 PMC6647859

[49] Y. van de Wal , Y. Kooy , P. van Veelen , S. Peña , L. Mearin , G. Papadopoulos , F. Koning , J. Immunol. 1998, 161, 1585. 9712018

[50] a) Y. Zhai , L. Chen , Q. Zhao , Z. H. Zheng , Z. N. Chen , H. Bian , X. Yang , H. Y. Lu , P. Lin , X. Chen , R. Chen , H. Y. Sun , L. N. Fan , K. Zhang , B. Wang , X. X. Sun , Z. Feng , Y. M. Zhu , J. S. Zhou , S. R. Chen , T. Zhang , S. Y. Chen , J. J. Chen , K. Zhang , Y. Wang , Y. Chang , R. Zhang , B. Zhang , L. J. Wang , X. M. Li , et al., Science 2023, 379, abg2482;

[51] C. Diskin , T. A. J. Ryan , L. A. J. O'Neill , Immunity 2021, 54, 19. 33220233 10.1016/j.immuni.2020.09.014

[52] J. S. Kang , L. B. Nam , O. K. Yoo , Y. S. Keum , Biochem. Pharmacol. 2020, 177, 114002. 32360363 10.1016/j.bcp.2020.114002

[53] Z. Zhang , C. Chen , F. Yang , Y. X. Zeng , P. Sun , P. Liu , X. Li , Mol. Cell 2022, 82, 2844. 35662396 10.1016/j.molcel.2022.05.009

[54] X. Shi , H. Zhou , J. Wei , W. Mo , Q. Li , X. Lv , Redox Biol. 2022, 58, 102553. 36459716 10.1016/j.redox.2022.102553 PMC9713374

[55] C. Su , T. Cheng , J. Huang , T. Zhang , H. Yin , Cell Rep. 2023, 42, 113040. 37624697 10.1016/j.celrep.2023.113040

[56] W. Li , Y. Li , J. Kang , H. Jiang , W. Gong , L. Chen , C. Wu , M. Liu , X. Wu , Y. Zhao , J. Ren , Cell Rep. 2023, 42, 112145. 36862550 10.1016/j.celrep.2023.112145

[57] M. C. Runtsch , S. Angiari , A. Hooftman , R. Wadhwa , Y. Zhang , Y. Zheng , J. S. Spina , M. C. Ruzek , M. A. Argiriadi , A. F. McGettrick , R. S. Mendez , A. Zotta , C. G. Peace , A. Walsh , R. Chirillo , E. Hams , P. G. Fallon , R. Jayamaran , K. Dua , A. C. Brown , R. Y. Kim , J. C. Horvat , P. M. Hansbro , C. Wang , L. A. J. O'Neill , Cell Metab. 2022, 34, 487. 35235776 10.1016/j.cmet.2022.02.002

[58] B. R. Sharma , T. D. Kanneganti , Nat. Immunol. 2021, 22, 550. 33707781 10.1038/s41590-021-00886-5 PMC8132572

[59] A. Hooftman , S. Angiari , S. Hester , S. E. Corcoran , M. C. Runtsch , C. Ling , M. C. Ruzek , P. F. Slivka , A. F. McGettrick , K. Banahan , M. M. Hughes , A. D. Irvine , R. Fischer , L. A. J. O'Neill , Cell Metab. 2020, 32, 468. 32791101 10.1016/j.cmet.2020.07.016 PMC7422798

[60] M. Certo , C. H. Tsai , V. Pucino , P. C. Ho , C. Mauro , Nat. Rev. Immunol. 2021, 21, 151. 32839570 10.1038/s41577-020-0406-2

[61] F. Jing , J. Zhang , H. Zhang , T. Li , Biol. Rev. Camb. Philos. Soc. 2025, 100, 172. 39279350 10.1111/brv.13135

[62] D. Zhang , Z. Tang , H. Huang , G. Zhou , C. Cui , Y. Weng , W. Liu , S. Kim , S. Lee , M. Perez‐Neut , J. Ding , D. Czyz , R. Hu , Z. Ye , M. He , Y. G. Zheng , H. A. Shuman , L. Dai , B. Ren , R. G. Roeder , L. Becker , Y. Zhao , Nature 2019, 574, 575. 31645732 10.1038/s41586-019-1678-1 PMC6818755

[63] a) Z. Zong , F. Xie , S. Wang , X. Wu , Z. Zhang , B. Yang , F. Zhou , Cell 2024, 187, 2375; 38653238 10.1016/j.cell.2024.04.002

[64] A. De Leo , A. Ugolini , X. Yu , F. Scirocchi , D. Scocozza , B. Peixoto , A. Pace , L. D'Angelo , J. K. C. Liu , A. B. Etame , A. Rughetti , M. Nuti , A. Santoro , M. A. Vogelbaum , J. R. Conejo‐Garcia , P. C. Rodriguez , F. Veglia , Immunity 2024, 57, 1105. 38703775 10.1016/j.immuni.2024.04.006 PMC11114377

[65] V. Zecchini , V. Paupe , I. Herranz‐Montoya , J. Janssen , I. M. N. Wortel , J. L. Morris , A. Ferguson , S. R. Chowdury , M. Segarra‐Mondejar , A. S. H. Costa , G. C. Pereira , L. Tronci , T. Young , E. Nikitopoulou , M. Yang , D. Bihary , F. Caicci , S. Nagashima , A. Speed , K. Bokea , Z. Baig , S. Samarajiwa , M. Tran , T. Mitchell , M. Johnson , J. Prudent , C. Frezza , Nature 2023, 615, 499. 36890229 10.1038/s41586-023-05770-w PMC10017517

[66] J. Cheng , J. Yan , Y. Liu , J. Shi , H. Wang , H. Zhou , Y. Zhou , T. Zhang , L. Zhao , X. Meng , H. Gong , X. Zhang , H. Zhu , P. Jiang , Cell Metab. 2023, 35, 961. 37178684 10.1016/j.cmet.2023.04.017

[67] M. D. Kornberg , P. Bhargava , P. M. Kim , V. Putluri , A. M. Snowman , N. Putluri , P. A. Calabresi , S. H. Snyder , Science 2018, 360, 449. 29599194 10.1126/science.aan4665 PMC5924419

[68] F. Humphries , L. Shmuel‐Galia , N. Ketelut‐Carneiro , S. Li , B. Wang , V. V. Nemmara , R. Wilson , Z. Jiang , F. Khalighinejad , K. Muneeruddin , S. A. Shaffer , R. Dutta , C. Ionete , S. Pesiridis , S. Yang , P. R. Thompson , K. A. Fitzgerald , Science 2020, 369, 1633. 32820063 10.1126/science.abb9818 PMC8744141

[69] a) H. J. Forman , H. Zhang , Nat. Rev. Drug Discov. 2021, 20, 689; 34194012 10.1038/s41573-021-00233-1 PMC8243062

[70] K. M. Holmström , T. Finkel , Nat. Rev. Mol. Cell Biol. 2014, 15, 411. 24854789 10.1038/nrm3801

[71] Y. Guo , Y. Liu , S. Zhao , W. Xu , Y. Li , P. Zhao , D. Wang , H. Cheng , Y. Ke , X. Zhang , Nat. Commun. 2021, 12, 7094. 34876574 10.1038/s41467-021-27428-9 PMC8651733

[72] a) Arthritis Rheumatol. 2015, 67, 1;

[73] J. Jia , A. Arif , B. Willard , J. D. Smith , D. J. Stuehr , S. L. Hazen , P. L. Fox , Mol. Cell 2012, 47, 656. 22771119 10.1016/j.molcel.2012.06.006 PMC3635105

[74] M. Battistella , W. Cuccuini , M. Elbouchtaoui , C. Leboeuf , L.‐F. Plassa , F. Bouhidel , A. Rigolet , V. Meignin , G. Socié , P. Ratajczak , A. Janin , J. Invest. Dermatol. 2014, 134, 864. 24005049 10.1038/jid.2013.371

[75] Y. Ji , X. Han , W. Tian , Y. Gao , S. Jin , L. Zhang , H. Shang , Vaccine 2018, 36, 7700. 30389191 10.1016/j.vaccine.2018.10.084

[76] F. Tokunaga , H. Nishimasu , R. Ishitani , E. Goto , T. Noguchi , K. Mio , K. Kamei , A. Ma , K. Iwai , O. Nureki , EMBO J. 2012, 31, 3856. 23032187 10.1038/emboj.2012.241 PMC3463848

[77] a) T. Jenuwein , C. D. Allis , Science 2001, 293, 1074; 11498575 10.1126/science.1063127

[78] a) K. Ishigaki , K. A. Lagattuta , Y. Luo , E. A. James , J. H. Buckner , S. Raychaudhuri , Nat. Genet. 2022, 54, 393; 35332318 10.1038/s41588-022-01032-z PMC9010379

[79] J.‐E. Park , R. A. Botting , C. Domínguez Conde , D.‐M. Popescu , M. Lavaert , D. J. Kunz , I. Goh , E. Stephenson , R. Ragazzini , E. Tuck , A. Wilbrey‐Clark , K. Roberts , V. R. Kedlian , J. R. Ferdinand , X. He , S. Webb , D. Maunder , N. Vandamme , K. T. Mahbubani , K. Polanski , L. Mamanova , L. Bolt , D. Crossland , F. de Rita , A. Fuller , A. Filby , G. Reynolds , D. Dixon , K. Saeb‐Parsy , S. Lisgo , et al., Science 2020, 367, ay3224. 10.1126/science.aay3224 PMC7611066 32079746

[80] R. Hohlfeld , K. V. Toyka , L. L. Miner , S. L. Walgrave , B. M. Conti‐Tronconi , J. Clin. Invest. 1988, 81, 657. 2449458 10.1172/JCI113369 PMC442511

[81] V. H. Engelhard , M. Altrich‐Vanlith , M. Ostankovitch , A. L. Zarling , Curr. Opin. Immunol. 2006, 18, 92. 16343885 10.1016/j.coi.2005.11.015

[82] a) A. L. Zarling , J. M. Polefrone , A. M. Evans , L. M. Mikesh , J. Shabanowitz , S. T. Lewis , V. H. Engelhard , D. F. Hunt , Proc. Natl. Acad. Sci. USA 2006, 103, 14889; 17001009 10.1073/pnas.0604045103 PMC1595446

[83] Y. Patskovsky , A. Natarajan , L. Patskovska , S. Nyovanie , B. Joshi , B. Morin , C. Brittsan , O. Huber , S. Gordon , X. Michelet , F. Schmitzberger , R. B. Stein , M. A. Findeis , A. Hurwitz , M. Van Dijk , E. Chantzoura , A. S. Yague , D. P. Smith , J. S. Buell , D. Underwood , M. Krogsgaard , Nat. Commun. 2023, 14, 3763. 37353482 10.1038/s41467-023-39425-1 PMC10290117

[84] F. Mohammed , D. H. Stones , A. L. Zarling , C. R. Willcox , J. Shabanowitz , K. L. Cummings , D. F. Hunt , M. Cobbold , V. H. Engelhard , B. E. Willcox , Oncotarget 2017, 8, 54160. 28903331 10.18632/oncotarget.16952 PMC5589570

[85] a) A. Alpízar , F. Marino , A. Ramos‐Fernández , M. Lombardía , A. Jeko , F. Pazos , A. Paradela , C. Santiago , A. J. R. Heck , M. Marcilla , Mol. Cell. Proteomics 2017, 16, 181; 27920218 10.1074/mcp.M116.063800 PMC5294207

[86] Y. Patskovsky , A. Natarajan , L. Patskovska , S. Nyovanie , B. Joshi , B. Morin , C. Brittsan , O. Huber , S. Gordon , X. Michelet , F. Schmitzberger , R. B. Stein , M. A. Findeis , A. Hurwitz , M. Van Dijk , E. Chantzoura , A. S. Yague , D. P Smith , J. S. Buell , D. Underwood , M. Krogsgaard , Nat. Commun. 2023, 14, 3763. 37353482 10.1038/s41467-023-39425-1 PMC10290117

[87] A. L. Zarling , S. B. Ficarro , F. M. White , J. Shabanowitz , D. F. Hunt , V. H. Engelhard , J. Exp. Med. 2000, 192, 1755. 11120772 10.1084/jem.192.12.1755 PMC2213507

[88] M. Cobbold , H. De La Peña , A. Norris , J. M. Polefrone , J. Qian , A. M. English , K. L. Cummings , S. Penny , J. E. Turner , J. Cottine , J. G. Abelin , S. A. Malaker , A. L. Zarling , H. W. Huang , O. Goodyear , S. D. Freeman , J. Shabanowitz , G. Pratt , C. Craddock , M. E. Williams , D. F. Hunt , V. H. Engelhard , Sci. Transl. Med. 2013, 5, 203ra125, 10.1126/scitranslmed.3006061 PMC4071620 24048523

[89] F. Marino , G. P. M. Mommen , A. Jeko , H. D. Meiring , J. A. M. van Gaans‐van den Brink , R. A. Scheltema , C. A. C. M. van Els , A. J. R. Heck , J. Proteome Res. 2016, 16, 34. 27503676 10.1021/acs.jproteome.6b00528

[90] T. Uhlmann , V. L. Geoghegan , B. Thomas , G. Ridlova , D. C. Trudgian , O. Acuto , Mol. Cell. Proteomics 2012, 11, 1489. 22865923 10.1074/mcp.M112.020743 PMC3494207

[91] J. Yagüe , J. Vázquez , J. A. L. D. Castro , Protein Sci. 2008, 9, 2210.

[92] F. Marino , G. P. Mommen , A. Jeko , H. D. Meiring , J. A. van Gaans‐van den Brink , R. A. Scheltema , C. A. van Els , A. J. Heck , J. Proteome Res. 2017, 16, 34. 27503676 10.1021/acs.jproteome.6b00528

[93] C. Slawson , G. W. Hart , Nat. Rev. Cancer 2011, 11, 678. 21850036 10.1038/nrc3114 PMC3291174

[94] a) P. Zhou , W. Y. Chang , D. A. Gong , J. Xia , W. Chen , L. Y. Huang , R. Liu , Y. Liu , C. Chen , K. Wang , N. Tang , A. L. Huang , Cell Metab. 2023, 35, 1961; 37797623 10.1016/j.cmet.2023.09.009

[95] a) J. C. Chatham , J. Zhang , A. R. Wende , Physiol. Rev. 2021, 101, 427; 32730113 10.1152/physrev.00043.2019 PMC8428922

[96] S. A. Malaker , S. A. Penny , L. G. Steadman , P. T. Myers , J. C. Loke , M. Raghavan , D. L. Bai , J. Shabanowitz , D. F. Hunt , M. Cobbold , Cancer Immunol. Res. 2017, 5, 376. 28314751 10.1158/2326-6066.CIR-16-0280 PMC5508727

[97] X. Wu , M. Xu , M. Geng , S. Chen , P. J. Little , S. Xu , J. Weng , Signal Transduct. Target Ther. 2023, 8, 220. 37244925 10.1038/s41392-023-01439-y PMC10224996

[98] M. H. Smith , J. R. Berman , J. Am. Med. Assoc. 2022, 327, 1194. 10.1001/jama.2022.0786 35315883

[99] A. J. Ytterberg , V. Joshua , G. Reynisdottir , N. K. Tarasova , D. Rutishauser , E. Ossipova , A. Haj Hensvold , A. Eklund , C. M. Sköld , J. Grunewald , V. Malmström , P. J. Jakobsson , J. Rönnelid , L. Padyukov , R. A. Zubarev , L. Klareskog , A. I. Catrina , Ann. Rheum. Dis. 2015, 74, 1772. 24817415 10.1136/annrheumdis-2013-204912

[100] A. M. Curran , A. A. Girgis , Y. Jang , J. D. Crawford , M. A. Thomas , R. Kawalerski , J. Coller , C. O. Bingham 3rd , C. H. Na , E. Darrah , Nat. Commun. 2023, 14, 1061. 36828807 10.1038/s41467-023-36620-y PMC9958131

[101] L. Santambrogio , Science 2023, 379, 1092. 36927028 10.1126/science.adg3925

[102] D. M. Lee , P. H. Schur , Ann. Rheum. Dis. 2003, 62, 870. 12922961 10.1136/ard.62.9.870 PMC1754666

[103] E. Pieterse , J. Hofstra , J. Berden , M. Herrmann , J. Dieker , J. van der Vlag , Clin. Exp. Immunol. 2015, 179, 68. 24758196 10.1111/cei.12359 PMC4260898

[104] R. C. Garduno , J. Dabritz , Front. Immunol. 2021, 12, 738762. 34707610 10.3389/fimmu.2021.738762 PMC8542854

[105] M. Nakayama , A. W. Michels , Front. Immunol. 2021, 12, 777788. 34868047 10.3389/fimmu.2021.777788 PMC8635517

[106] Y. Zhai , P. Zhu , Clin. Transl. Med. 2023, 13, 1373. 10.1002/ctm2.1373 PMC10435718 37592373

[107] N. Xie , G. Shen , W. Gao , Z. Huang , C. Huang , L. Fu , Signal Transd. Targeted Ther. 2023, 8, 9. 10.1038/s41392-022-01270-x PMC9816309 36604431

[108] D. J. Baker , Z. Arany , J. A. Baur , J. A. Epstein , C. H. June , Nature 2023, 619, 707. 37495877 10.1038/s41586-023-06243-w PMC12522170

[109] M. D. Jyothi , R. A. Flavell , T. L. Geiger , Nat. Biotechnol. 2002, 20, 1215. 12426577 10.1038/nbt758

[110] J. Yi , A. T. Miller , A. S. Archambault , A. J. Jones , T. R. Bradstreet , S. Bandla , Y. S. Hsu , B. T. Edelson , Y. W. Zhou , D. H. Fremont , T. Egawa , N. Singh , G. F. Wu , C. S. Hsieh , Sci. Immunol. 2022, 7, abo0777. 10.1126/sciimmunol.abo0777 PMC9867937 36206355

[111] S. Fishman , M. D. Lewis , L. K. Siew , E. De Leenheer , D. Kakabadse , J. Davies , D. Ziv , A. Margalit , N. Karin , G. Gross , F. S. Wong , Mol. Ther. 2017, 25, 456. 28109957 10.1016/j.ymthe.2016.12.007 PMC5368593

[112] a) H. R. MacDonald , R. Schneider , R. K. Lees , R. C. Howe , H. Acha‐Orbea , H. Festenstein , R. M. Zinkernagel , H. Hengartner , Nature 1988, 332, 40; 3126397 10.1038/332040a0

[113] N. M. Gonzalez , D. Zou , Z. Zeng , F. X. Feng , X. Zhang , C. Sannes , A. Gu , Y. Zu , W. Chen , Immunology 2025, 174, 239. 39648274 10.1111/imm.13881

[114] Z. Wang , R. Li , W. Fu , H. Cheng , Y. Zhang , G. Tang , J. Yang , J. Wang , X. Ni , Front. Immunol. 2024, 15, 1460687. 39776911 10.3389/fimmu.2024.1460687 PMC11703850

[115] O. V. Britanova , K. R. Lupyr , D. B. Staroverov , I. A. Shagina , A. A. Aleksandrov , Y. Y. Ustyugov , D. V. Somov , A. Klimenko , N. A. Shostak , I. V. Zvyagin , A. V. Stepanov , E. M. Merzlyak , A. N. Davydov , M. Izraelson , E. S. Egorov , E. A. Bogdanova , A. K. Vladimirova , P. A. Iakovlev , D. A. Fedorenko , R. A. Ivanov , V. I. Skvortsova , S. Lukyanov , D. M. Chudakov , Nat. Med. 2023, 29, 2731. 37872223 10.1038/s41591-023-02613-z PMC10667094

[116] X. Jin , T. Dong , Q. Wang , Y. Xie , X. Fang , C. Wei , S. Liu , X. Zheng , P. Wang , D. Zhu , L. Cao , S. Dong , K. Fang , C. Zhong , J. Wang , F. Hu , Z. Li , Sci. Bull (Beijing) 2024, 69, 2920. 39153908 10.1016/j.scib.2024.02.042

[117] A. Ben‐Nun , H. Wekerle , I. R. Cohen , Nature 1981, 292, 60. 6974307 10.1038/292060a0

[118] D. Karussis , H. Shor , J. Yachnin , N. Lanxner , M. Amiel , K. Baruch , Y. Keren‐Zur , O. Haviv , M. Filippi , P. Petrou , S. Hajag , U. Vourka‐Karussis , A. Vaknin‐Dembinsky , S. Khoury , O. Abramsky , H. Atlan , I. R. Cohen , R. Abulafia‐Lapid , PLoS One 2012, 7, 50478. 10.1371/journal.pone.0050478 PMC3522721 23272061

[119] R. N. Cotton , M. Wegrecki , T. Y. Cheng , Y. L. Chen , N. Veerapen , J. Le Nours , D. P. Orgill , B. Pomahac , S. G. Talbot , R. Willis , J. D. Altman , A. de Jong , I. Van Rhijn , R. A. Clark , G. S. Besra , G. Ogg , J. Rossjohn , D. B. Moody , J. Exp. Med. 2021, 218, 20202699. 10.1084/jem.20202699 PMC8111460 33961028

[120] a) J. H. Kim , Y. Hu , T. Yongqing , J. Kim , V. A. Hughes , J. Le Nours , E. A. Marquez , A. W. Purcell , Q. Wan , M. Sugita , J. Rossjohn , F. Winau , Nat. Immunol. 2016, 17, 1159; 27548435 10.1038/ni.3523 PMC5791155

[121] B. D. Evavold , J. Sloan‐Lancaster , P. M. Allen , Immunol. Today 1993, 14, 602. 8305133 10.1016/0167-5699(93)90200-5

[122] M. Candia , B. Kratzer , W. F. Pickl , Int. Archives Allergy Immunol. 2016, 170, 211. 10.1159/000448756 PMC7058415 27642756

[123] a) N. Lorenzo , A. Barberá , M. C. Domínguez , A. M. Torres , M. V. Hernandez , I. Hernandez , R. Gil , J. Ancizar , H. Garay , O. Reyes , F. Altruda , L. Silengo , G. Padrón , Autoimmunity 2012, 45, 449; 22686732 10.3109/08916934.2012.697592

[124] a) L. K. Myers , B. Tang , E. F. Rosioniec , J. M. Stuart , A. H. Kang , Critical Rev. Immunol. 2007, 27, 345; 18197813 10.1615/critrevimmunol.v27.i4.40

